# A Genome-Wide Scan for MicroRNA-Related Genetic Variants Associated With Primary Open-Angle Glaucoma

**DOI:** 10.1167/iovs.17-22410

**Published:** 2017-10

**Authors:** Mohsen Ghanbari, Adriana I. Iglesias, Henriët Springelkamp, Cornelia M. van Duijn, M. Arfan Ikram, Abbas Dehghan, Stefan J. Erkeland, Caroline C. W. Klaver, Magda A. Meester-Smoor, for the International Glaucoma Genetics Consortium (IGGC)

**Affiliations:** 1 Department of Epidemiology, Erasmus University Medical Center, Rotterdam, The Netherlands; 2 Department of Genetics, Mashhad University of Medical Sciences, Mashhad, Iran; 3 Department of Ophthalmology, Erasmus University Medical Center, Rotterdam, The Netherlands; 4 Department of Neurology, Erasmus University Medical Center, Rotterdam, The Netherlands; 5 Department of Epidemiology & Biostatistics, Imperial College London, London, United Kingdom; 6 Department of Immunology, Erasmus University Medical Center, Rotterdam, The Netherlands; 7 Department of Ophthalmology, Radbound University Nijmegen Medical Centre, Nijmegen, The Netherlands

**Keywords:** primary open-angle glaucoma (POAG), microRNA, genetic variant, GWAS

## Abstract

**Purpose:**

To identify microRNAs (miRNAs) involved in primary open-angle glaucoma (POAG), using genetic data. MiRNAs are small noncoding RNAs that posttranscriptionally regulate gene expression. Genetic variants in miRNAs or miRNA-binding sites within gene 3′-untranslated regions (3′UTRs) are expected to affect miRNA function and contribute to disease risk.

**Methods:**

Data from the recent genome-wide association studies on intraocular pressure, vertical cup-to-disc ratio (VCDR), cupa area and disc area were used to investigate the association of miRNAs with POAG endophenotypes. Putative targets of the associated miRNAs were studied according to their association with POAG and tested in cell line by transfection experiments for regulation by the miRNAs.

**Results:**

Of 411 miRNA variants, rs12803915:A/G in the terminal loop of pre–miR-612 and rs2273626:A/C in the seed sequence of miR-4707 were significantly associated with VCDR and cup area (*P* values < 1.2 × 10^−4^). The first variant is demonstrated to increase the miR-612 expression. We showed that the second variant does not affect the miR-4707 biogenesis, but reduces the binding of miR-4707-3p to *CARD10*, a gene known to be involved in glaucoma. Moreover, of 72,052 miRNA-binding-site variants, 47 were significantly associated with four POAG endophenotypes (*P* value < 6.9 × 10^−6^). Of these, we highlighted 10 variants that are more likely to affect miRNA-mediated gene regulation in POAG. These include rs3217992 and rs1063192, which have been shown experimentally to affect miR-138-3p– and miR-323b-5p–mediated regulation of *CDKN2B*.

**Conclusions:**

We identified a number of miRNAs that are associated with POAG endophenotypes. The identified miRNAs and their target genes are candidates for future studies on miRNA-related therapies for POAG.

Primary open-angle glaucoma (POAG), the most common optic neuropathy, is the leading cause of irreversible blindness, affecting approximately 60 million individuals worldwide.^1,2^ The disease is characterized by progressive loss of retinal ganglion cells and optic nerve degeneration that can be secondary to elevated intraocular pressure (IOP).^3^ The optic nerve damage is characterized by an increase in cup size, which is the central area of the optic disc. Cup enlargement can be measured by the vertical cup-to-disc ratio (VCDR), comparing the vertical diameter of the cup with vertical diameter of the total optic disc.^4^ The VCDR ranges from 0 to 1; a ratio above 0.7 or an asymmetry between eyes above 0.2 is considered as suspect for glaucoma in the clinical setting.^5^ POAG is presumed to be a complex progressive neurodegenerative disorder caused by multiple genetic as well as environmental factors.^2^ Previous genome-wide association studies (GWASs) have revealed a number of susceptibility loci for POAG by studying the disease directly or its endophenotypes including IOP and optic disc parameters (VCDR, cup area, and disc area).^5,6^ Most of the associated variants identified by GWASs are located in noncoding regions of the genome and their mechanistic contributions to POAG and its endophenotypes remain poorly understood.^5,6^

MicroRNAs (miRNAs) are small noncoding RNAs, consisting of 19 to 22 nucleotides, that posttranscriptionally regulate gene expression.^7^ There are strong indications that miRNAs play important roles in the pathogenesis of POAG.^8–11^ For example, miR-29b and miR-24 are involved in gene regulation in trabecular meshwork cells.^12^ Moreover, the miRNA expression levels have been linked to maintaining the balance of the aqueous humor, the change in the trabecular meshwork, and the apoptosis of the retinal ganglion cells.^13–15^ A number of miRNAs (e.g., miR-200c, miR-204, miR-183, and miR-182) are also reported as potential diagnostic biomarkers or therapeutic targets for glaucoma.^9,16^ The biogenesis of miRNAs is a multistep coordinated process.^17,18^ In the nucleus, miRNA genes are initially transcribed as long primary transcripts. Further processing and cleavage by the RNase *Drosha* and *Dicer* enzymes generate mature miRNAs.^17,18^ The mature miRNAs are subsequently incorporated into the RNA-induced silencing complex (RISC) to interact with the 3′-untranslated region (3′UTR) of target mRNAs, resulting in mRNA degradation or translational repression.^7,18^ Genetic variants in miRNA-encoding sequences can have profound effects on miRNA biogenesis and function.^19,20^ In addition, polymorphisms located in miRNA-binding sites within the 3′UTR of target genes are expected to affect miRNA-mediated gene regulation.^20,21^ Previous studies^19,21–23^ have shown that such miRNA-related variants contribute to complex disease risk; a candidate variant association study^24^ has also recently reported the association between genetic variation in miR-182 and POAG. In this study, we applied an in silico study on the existing GWAS of IOP and optic disc parameters^5^ and performed in vitro experiments to identify miRNAs and target genes that may play a role in POAG.

## Methods

### Genome-Wide Association Studies on Glaucoma Endophenotypes

To examine the association of miRNA-related genetic variants with POAG endophenotypes, we used data from the recent GWAS on glaucoma endophenotypes provided by the International Glaucoma Genetics Consortium (IGGC).^5^ Characteristics of the IGGC have been described elsewhere.^5^ In brief, the IGGC has conducted genome-wide association meta-analyses on four glaucoma endophenotypes including IOP (*n* = 37,930 individuals), VCDR (*n* = 23,899 individuals), cup area (*n* = 22,489 individuals), and disc area (*n* = 22,504 individuals). The IGGC, using imputation to the 1000 Genomes (1000G) reference panel, has tested the association of approximately 8 million single-nucleotide polymorphisms (SNPs) with minor allele frequency (MAF) > 0.01 with glaucoma endophenotypes. The summary association statistics for these phenotypes are available in the public domain (http://www.dropbox.com/sh/3j2h9qdbzjwvaj1/AABFD1eyNetiF63I5bQooYura?dl=0).

### Genetic Variants Located in miRNAs and miRNA-Binding Sites

**Figure 1:**
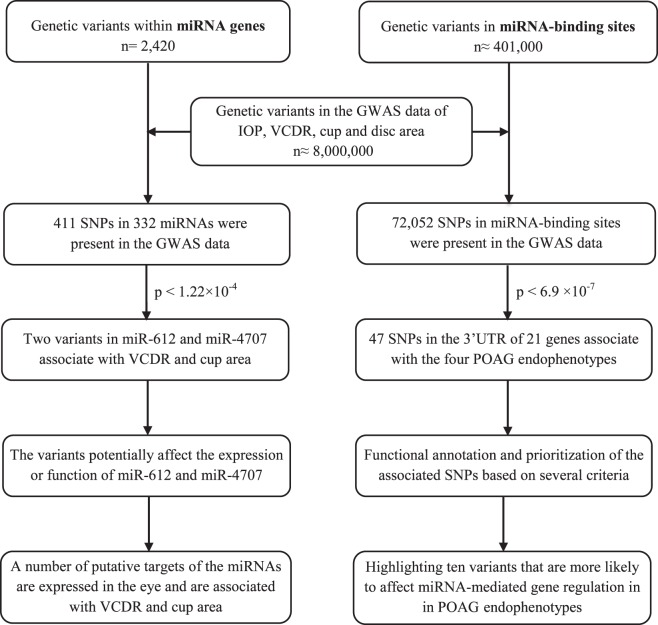
A schematic workflow of our analyses to identify genetic variants in miRNAs as well as in miRNA-binding sites within gene 3′UTRs that are associated with four POAG endophenotypes.

A total of 2420 genetic variants located in miRNA-encoding sequences were retrieved from miRNASNP v2 (database update April 2015)^25^ and literature review (searching for miRNA genetic variants in PubMed). These include variants in precursor miRNAs (60–80 nucleotides [nt]), mature miRNA sequences (19–22 nt), and miRNA seed regions (nucleotides 2–8 from the 5′-end of a mature miRNA, which is defined as the most crucial part of an miRNA for target recognition and has to be perfectly complementary to the binding site of a target mRNA). Of 2420 miRNA variants, 411 (in 332 miRNAs) were available in the GWAS data of glaucoma endophenotypes and were selected for association analysis. These variants include 283 in pre-miRNA loci, 87 in mature miRNA sequences, and 41 in miRNA seed regions. Moreover, we extracted almost 401,000 genetic variants (including SNPs and INDELs) in miRNA-binding sites within the gene 3′UTRs by using PolymiRTS v3 (database update October 2014).^26^ Of these, 72,052 miRNA-binding-site variants were available in the GWAS data of glaucoma endophenotypes and were included for association analysis. The Bonferroni correction was used to adjust the *P* value for multiple comparisons. To this end, we divided the critical *P* value (*α* = 0.05) by the number of genetic variants in each group (miRNAs and miRNA-binding sites). As we studied 411 variants in miRNAs, the significance threshold was set on 1.22 × 10^−4^ (0.05/411) for miRNA genetic variants. Likewise, the significance threshold for genetic variants in miRNA-binding sites was set on 6.94 × 10^−7^ (0.05/72,052), as we studied 72,052 variants in this group (Fig. 1). Regional association plots showing the association of miRNA-related variants with the studied phenotypes were made by using LocusZoom.^27^

### The Effect of Variants on the miRNA Secondary Structure and Expression

The secondary structure of pre-miRNA is critical for the miRNA biogenesis.^28^ The Vienna RNAfold algorithm (ViennaRNA package 2.0) was used to predict the impact of variants in miRNAs on the hairpin stem-loop structure of pre-miRNAs.^29^ This program calculates the changes in minimum free energy (MFE) of the thermodynamic ensemble of the hairpin structure of miRNA (wild type and mutant). Next, to test the functional impact of an miRNA variant on the expression of the mature miRNA, we cloned the pre-miRNA sequence containing either the major or minor allele behind the gene encoding green fluorescent protein (GFP) in the expression plasmid MSCV-BC (Murine Stem Cell Virus-Bar Coded), resulting in GFP-miRNA fusion transcripts as described previously.^30^ For cloning purposes, the restriction enzyme sites *Xho*I and *Eco*RI were inserted in forward and reverse primers, respectively. The primers are listed in Supplementary Table S1. We used Sanger sequencing to validate the inserts of all constructs. HEK293 cells were plated at a density of 5 × 10^5^ cells per well in a six-well plate and were cultured in 2 mL Dulbecco's modified Eagle's medium (DMEM) containing 10% fetal bovine serum and 100 units/mL penicillin/streptomycin in a 37°C incubator with 5% CO_2_. The next day, cells were transiently transfected with 1 μg GFP-miRNA constructs containing either the major or minor allele by using LipofectamineR RNAiMAX (Invitrogen, Carlsbad, CA, USA) according to the manufacturer's instructions. After 48 hours, total RNA was isolated with the TRIZOL reagent (Invitrogen) according to the manufacturer's protocols. The concentration and purity of RNA samples were determined with a NanoDrop ND-1000 spectrophotometer (NanoDrop, Wilmington, DE, USA). TaqMan Assays (Applied Biosystems, Foster City, CA, USA) were used to measure the expression levels of GFP and miRNA by quantitative RT-PCR. The expression levels of mutant and wild-type miRNA relative to GFP were calculated as previously described.^30^ RNU6B was used as an internal loading control for miRNA expression. All experiments were performed in triplicate and repeated at least three times.

### Association of miRNA Target Genes With Glaucoma Endophenotypes

The biological role of miRNAs is dictated through regulating expression of their target mRNAs. We aimed to identify target genes that may mediate the downstream effects of the associated miRNAs in relation to POAG endophenotypes. Since such genes should be also associated with the studied phenotypes, we used the GWAS data in a candidate gene approach and searched among the putative target genes of an miRNA for those that were associated with POAG endophenotypes. To this end, we extracted the miRNA putative targets by using two commonly used online databases: TargetScan v7.0 (total context^++^ score > 0.1)^31^ and miRDB.^32^ TargetScan (http://www.TargetScan.org/; in the public domain) predicts biological targets of miRNAs by searching for the presence of conserved sites that match the seed region of each miRNA. The predictions in this database are ranked by the predicted efficacy of targeting as calculated by using cumulative context++ scores of the sites by considering several features to predict the most effective target mRNAs.^31^ The targets in miRDB (http://mirdb.org//; in the public domain) were predicted by a bioinformatics tool, which was developed by analyzing thousands of miRNA–target interactions from high-throughput sequencing experiments.^32^ The GWAS data from glaucoma endophenotypes were used to examine the association of genetic variants in the predicted miRNA target genes with the phenotype of interest.^5^ The Bonferroni correction was used to calculate the significance level based on the number of tested genetic variants in all predicted target genes of each miRNA.

### Interaction Analysis Between an miRNA and Its Target Genes Using the Rotterdam Study Data

The external validity of association between an miRNA variant and associated phenotype may improve when the variant affects the miRNA target genes involved in the phenotype. We thus tested the interaction between associated miRNA variants and the most significant variant in their related target genes in relation to POAG endophenotype, using the Rotterdam Study data. To examine the interaction, we introduced the statistical product of miRNA variant and target gene variant in the logistic regression model, adjusted for age and sex: POAG endophenotype ∼ age + sex + miRNA SNP + top SNP in target gene + (miRNA SNP × top SNP in target gene). The design of the Rotterdam Study has been described in detail elsewhere.^33^ The baseline characteristics of the subjects are shown in Supplementary Table S2.

### Luciferase Reporter Assays

A luciferase reporter assay system was used to examine the interaction between an miRNA and the 3′UTR of its putative target gene. In addition, this experiment was used to determine the impact of candidate variant on the miRNA–target gene interaction. To this end, primers were designed to amplify the 3′UTR sequence of the miRNA target gene and included restriction enzyme sites *Xba*I for the forward primer and *Apa*I for the reverse. The 3′UTR sequences containing putative binding site (wild type and mutated) of the miRNA were amplified and cloned into the pGL3 Luciferase reporter vector (Promega, Madison, WI, USA) downstream of the Luciferase open reading frame.^30^ All the primers are shown in Supplementary Table S3. The inserts of all constructs were confirmed by Sanger sequencing. HEK293 cells (*n* = 10,000) were plated into 96-well plates and cotransfected with 1 μg pGL3 containing the 3′UTR with either the major or minor allele, miRNA mimic (mirVana Mimics; Thermo Fischer Scientific, Waltham, MA, USA), and a plasmid expressing the Renilla luciferase that served as transfection control, with Lipofectamine RNAiMAX (Invitrogen). Luciferase activity was measured with the Dual-Glo Luciferase Assay System according to manufacturer's protocol (Promega). Renilla activity was used for normalization of the data. All experiments were performed in triplicate and repeated in three independent experiments.

### Functional Annotation of miRNA-Binding-Site Variants Associated With Glaucoma Endophenotypes

Specific criteria have been suggested to establish whether 3′UTR variants located in miRNA-binding sites may affect miRNA-mediated gene regulation.^20,21^ These include association between the variant and the phenotype of interest, expression of hosting gene and related miRNA in a relevant tissue, and an allele-specific regulation of the target transcript by miRNA.^20,21^ These criteria were used to prioritize the miRNA-binding-site variants that are more likely to be functional. We first retrieved proxy SNPs in high linkage disequilibrium (LD) (*R*^2^ threshold > 0.8, limit distance 100 kb, and population panel CEU) with the binding-site variants and checked their effects on protein structure, gene regulation, and splicing by using the HaploRegv4.1 (http://www.broadinstitute.org/mammals/haploreg/haploreg.php; in the public domain). The HaploReg 4.1 was also used to evaluate the functional potential of the LD variants associated with glaucoma endophenotypes. To scan the correlation between the identified variants and expression levels of the host transcripts, we used expression quantitative trait loci (cis-eQTL) data from the GTExV6 (http://www.gtexportal.org/home/; in the public domain) and Genenetwork (http://genenetwork.nl/bloodeqtlbrowser/; in the public domain). To check whether miRNAs in our collection are expressed in ocular tissues, two online databases were screened, miRetina^34^ and HMDD.^35^ Moreover, we used expression data from previous miRNA profiling studies in human ocular tissues, including ciliary body, cornea, and trabecular meshwork and aqueous humor.^13–15^ Other miRNA information, including miRNA conservation in different species, was obtained from miRBase (release 20).^36^ In addition, the Ocular Tissue Database (https://genome.uiowa.edu/otdb/; in the public domain) was used to examine the expression of the identified miRNA target genes across the eye tissues. Supplementary Table S4 shows a list of web tools and databases that we used for our analyses.

## Results

### Two miRNA Variants Were Associated With POAG Endophenotypes

We examined the association of 411 miRNA variants (in 332 miRNA genes) with IOP, VCDR, cup area, and disc area (Fig. 1). Two miRNA variants passed the Bonferroni-corrected significance threshold of 1.22 × 10^−4^ (0.05/411). These variants include rs12803915:A/G (Chr11:65444508) in the terminal loop of pre–miR-612 sequence associated with VCDR (*P* value = 4.6 × 10^−9^, *β* = −0.009) and cup area (*P* value = 1.2 × 10^−7^, *β* = −0.014), and rs2273626:A/C (Chr14:22956973) in the seed sequence of miR-4707 associated with VCDR (*P* value = 9.5 × 10^−5^, *β* = −0.005) and cup area (*P* value = 9.9 × 10^−5^, *β* = −0.008) (Table 1). No miRNA variants were significantly associated with IOP and disc area. Supplementary Table S5 shows genetic variants in miRNAs that are nominally associated (*P* value < 0.05) with POAG endophenotypes.

**Table 1:**
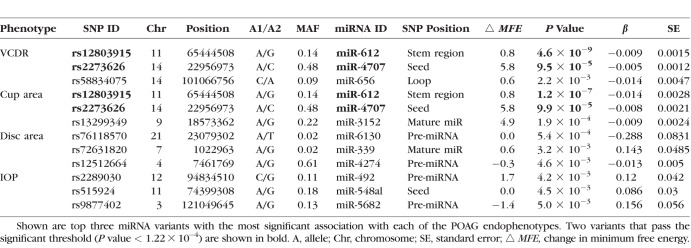
Association of Top miRNA Variants With POAG Endophenotypes

### The Impact of Variants on the miRNA Secondary Structure and Expression

**Figure 2:**
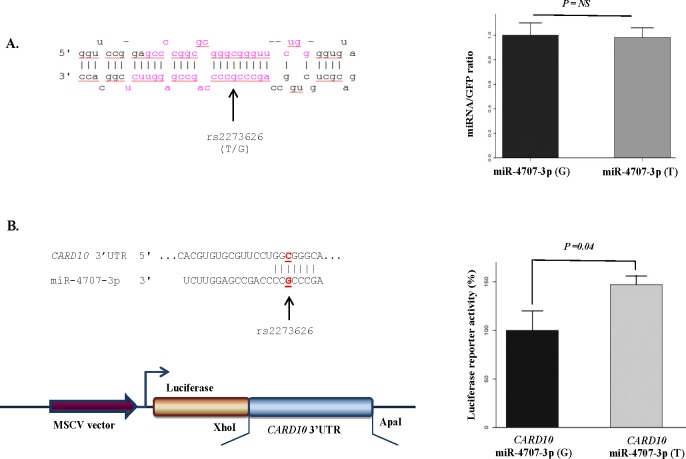
The impact of SNP rs2273626 in the seed sequence of miR-4707 on miRNA production and targeting. (A) The figure shows the predicted hairpin structure of miR-4707 containing rs2273626, which was associated with VCDR and cup area. The mature miRNA sequences (3p and 5p) are shown in red and the position of variants is depicted by an arrow. To examine the effect of rs2273626 on the miR-4707 expression level, HEK293 cells were transfected with GFP-miRNA transcripts containing either the minor allele T or the major allele G. The levels of mature miRNA relative to GFP transcript levels were calculated. (B) Luciferase reporter assays indicating miR-4707-3p–mediated repression of CARD10. HEK293 cells were cotransfected with CARD10 3′UTR luciferase reporter vector and GFP-miRNA transcripts containing either the minor allele T or the major allele G. This experiment indicates a significant difference (P = 0.04) between the relative luciferase activity of the CARD10 3′UTR construct in the presence of miR-4707-3p containing the major allele and the minor allele. Our results suggest that rs2273626 diminishes the regulatory interaction between miR-4707-3p and CARD10, resulting in increased CARD10 levels. All experiments were performed in triplicates and repeated at least three times. Error bars represent standard deviation (SD). NS, nonsignificant.

The impact of rs12803915 and rs2273626 on the miRNA biogenesis was measured by performing structural analysis of the miRNA hairpin, using Vienna RNAfold algorithm.^29^ We observed 0.8 kcal/mol difference in MFE of the thermodynamic ensemble of the miRNA hairpin structure for pre–miR-612 and 5.8 kcal/mol difference for pre–miR-4707, suggesting that the variants may affect their miRNA production (Supplementary Fig. S1). Rs12803915 (minor allele A) in pre–miR-612 has been demonstrated previously to increase expression of the mature miRNA in vitro.^37^ Additionally, we cloned the miR-4707 precursor with either the rs2273626 major allele G or the minor allele T behind GFP in the pMSCV-BC vector. HEK293 cells were transfected with the vectors expressing transcripts with GFP and pre-miRNA. We observed no significant difference between expression levels of mature miR-4707 in cells transfected with the pre-miRNA sequence containing the major and the minor allele (Fig. 2A). This indicates that the effect of rs2273626 on the miR-4707 biogenesis is minor and not detectable with this experimental setup.

### Target Genes of miR-612 and miR-4707 Associated With VCDR and Cup Area

To identify target genes that may mediate the downstream effect of miR-612 and miR-4707 in relation to VCDR and cup area, we studied the association of their putative target genes with these phenotypes, using the GWAS data.^5^ Supplementary Table S6 displays four predicted target genes of miR-612 and eight predicted target genes of miR-4707 that are associated with VCDR and cup area. Using data from the Ocular Tissue Database, we showed that these associated target genes are expressed in the eye tissues (Supplementary Table S7). We further tested the interaction between the identified miRNA variants and the top variant in their highlighted predicted target genes (associated with glaucoma endophenotypes and expressed in the eye), using the Rotterdam Study data. This analysis showed a significant interaction (*P* value = 0.028) between rs2273626 in miR-4707 and the top variant (rs6000755) in *CARD10* (Supplementary Table S8). Luciferase reporter assays then demonstrated that miR-4707-3p downregulates the expression of *CARD10* (Fig. 2B). Our experiments further showed a significant difference in luciferase activity of the *CARD10 3′UTR* construct in the presence of miR-4707-3p containing the major allele G, compared to the minor allele T at rs2273626 site (Fig. 2B). These data indicate that the minor allele G decreases the binding of miR-4707-3p to *CARD10*.

### Multiple miRNA-Binding-Site SNPs Were Associated With POAG Endophenotypes

In the second part of our study, we examined the associations of 72,052 miRNA-binding-site SNPs (available in the GWAS data^38^) with POAG endophenotypes (Supplementary Fig. S2). Of these, 47 SNPs (located in the 3′UTR of 21 genes) passed the Bonferroni-corrected significance threshold of 6.94 × 10^−7^, which are associated with one or more of the POAG endophenotypes. These SNPs are predicted to affect the putative interaction between their host genes and a number of miRNAs that are shown in Supplementary Table S9. To highlight the binding-site variants that are more likely to affect miRNA-mediated gene regulation, we prioritized them on the basis of various criteria (e.g., the strength of association, LD pattern, eQTL data, coexpression of miRNA and target gene in relevant tissue). Haploreg v4.1 data, we found showed that many of the binding-site SNPs (*n* = 37) have no nonsynonymous proxy variants in strong LD (*R*^2^ > 0.8) (Supplementary Table S10). The cis-eQTL data further showed that 33 of the SNPs are correlated with the expression levels of their genes in different tissues (Supplementary Table S10). The Ocular Tissue Database showed that all 21 hosting genes are expressed in the eye (Supplementary Table S11). Using the miRNA expression databases, we found evidence for the expression of several miRNAs in our collection in the eye (Supplementary Table S12). After prioritizing the 47 associated SNPs, based on their functional score, we highlighted 10 variants that are more likely to affect miRNA-mediated gene regulation in POAG (Table 2; Supplementary Fig. S3). These include two SNPs in the 3′UTR of *CDKN2B*, rs3217992 and rs1063192, which have been demonstrated previously to affect miR-138-3p– and miR-323b-5p–mediated regulation of *CDKN2B*.^21^

**Table 2:**
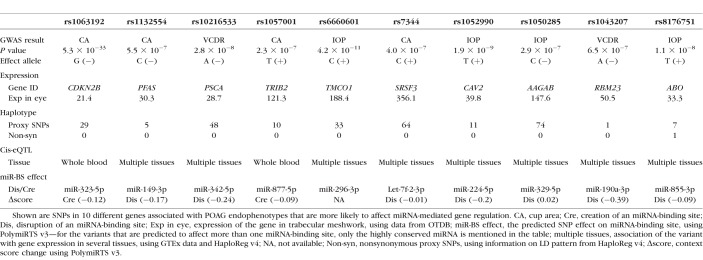
Characteristics of 10 3′UTR Variants Associated With POAG Endophenotypes Within miRNA-Binding Sites

## Discussion

In this study, we performed a genome-wide scan to identify miRNAs associated with POAG endophenotypes, using genetic data. We found genetic variants in the miR-612 precursor and in the miR-4707 seed region significantly associated with VCDR and cup area. The variant in miR-612 has been previously demonstrated to increase the miRNA expression.^37^ We showed that the variant in miR-4707 does not influence the miRNA expression, but affects the binding of miR-4707 to one of its glaucoma-associated target genes, *CARD10*. Moreover, we identified 47 SNPs in miRNA-binding sites (within the 3′UTR of 21 genes) that are significantly associated with POAG endophenotypes. After prioritization, we highlighted the 3′UTR variants that are more likely to affect miRNA-mediated gene regulation in POAG.

Several studies^8,9,15^ have shown previously the contribution of miRNAs to the pathophysiology of POAG. However, these studies are mainly focused on differentially expressed miRNAs indentified by miRNA profiling in a small number of samples, which makes it difficult to interpret their results to the general population. In addition, miRNA profiling studies are sometimes subject to confounding bias or reverse causation, and cannot provide evidence for a causal role of the identified miRNAs in relation to a disease. Here, we used a genetic approach that is proven to be efficient and successful for identification of miRNAs involved in complex traits.^19–23^ In this approach, the starting point is a linkage between miRNA variants and the trait of interest. The main advantage of the genetic approach compared to miRNA expression profiling is that when a variant is associated with disease risk, it supports the idea that the miRNA may have a primary effect in the disease pathogenesis. Given that genetic variants are randomly inherited, it is unlikely that other extrinsic factors (e.g., lifestyle) are associated with them. Moreover, as the DNA sequence is constant over the life course, reverse causation is also refuted. Another advantage of the genetic approach is that diseases with no accessible affected tissues (e.g., eye tissue of glaucoma patients) can still be studied, since genomic DNA is available from other accessible tissues such as blood. The main limitation of the genetic approach is that it is restricted to miRNAs with genetic variants available in the GWAS data. Therefore, both genetic and miRNA profiling approaches are valuable and can work complementarily to analyze different aspects of the role of miRNAs in disease pathogenesis. The genetic approach indicates whether miRNAs are potentially casual or risk factors for a disease and the miRNA profiling approach demonstrates whether the expression of the disease-associated miRNAs is altered in the affected tissue.

We found two variants in the precursor and seed region of miRNAs significantly associated with POAG endophenotypes. The first variant is rs12803915 located in miR-612. The GWAS data show that the variant minor allele A is associated with decreased VCDR and cup area.^5^ The variant minor allele has been demonstrated previously to increase the expression of mature miR-612 in vitro.^37^ The location of variant in the terminal loop of miR-612 possibly improves the pre-miRNA processing, and consequently increases the miRNA expression.^39–42^ Next, we highlighted four putative miR-612 targets (*USH2A, FAM101A, JRK*, and *SIX4)* that are also associated with VCDR and cup area and are expressed in the eye. These target genes might mediate the downstream effect of miR-612 in POAG. Together, our results suggest a protective role for miR-612 in relation to POAG. Future experimental studies are needed to further elucidate the role of miR-612 and its highlighted target genes in the pathogenesis of POAG.

The second associated variant is rs2273626 located in the seed sequence of miR-4707, an miRNA that is shown to be expressed in aqueous humor of glaucoma patients.^43^ The variant minor allele A was negatively associated with VCDR and cup area in the GWAS data.^5^ A polymorphism in an miRNA seed sequence is expected to strongly influence the miRNA activity and possibly risk of disease.^20,44^ We tested the two mechanisms through which a seed sequence variant may affect miR-4707 function. Our results showed that rs2273626 has no significant effect on the miRNA expression levels. However, the variant reduces the interaction between miR-4707-3p and *CARD10*, presumably resulting in higher *CARD10* levels. The *CARD10* gene is reported in several GWAS to be associated with glaucoma.^6,45–47^ The activity of *CARD10* has been suggested to have a neuroprotective role in glaucomatous optic neuropathy.^45^ POAG is a disease of enhanced retinal ganglion cell apoptosis.^48^ It has been postulated that *CARD10* controls optic disc area possibly through ganglion axonal survival via the activation of the NFκB signaling pathway.^46,49^ Overexpression of *CARD10* has been shown to be related with increased cell survival and proliferation.^50,51^ Conversely, homozygous knockout of *CARD10* in a mouse model can cause nonviability through neural tube defects.^52^ *CARD10* has been also shown to be required in neural crest cell survival through G protein–coupled receptor induction of NFκB activation.^52^ Retinal ganglion cell damage from NFκB is likely to be due to glial cell activation and IL-1β secretion.^53^ Given the intimate and complex relationship between *CARD10*, NFκB, and apoptosis,^47^ it is biologically plausible that lower levels of *CARD10* result in increased retinal ganglion cell apoptosis. In our study, the negative association between the miR-4707 variant and POAG may be explained in part by higher *CARD10* expression due to the disruption of miR-4707–mediated gene regulation.

In the second part of this study, we investigated the association of miRNA-binding-site variants with POAG endophenotypes and identified 47 associated SNPs. GWASs are based on tagging variants that are in high LD with functional variant(s) in the region; it is thus challenging to determine the exact localization of the variants that cause the associations. Here we prioritized the 47 associated SNPs on the basis of a set of predefined criteria (e.g., the strength of association, LD evaluation, eQTL analysis, coexpression of the miRNA and the target gene in relevant tissue).^20,21^ We highlighted 10 of the variants that are more likely to affect the putative miRNA-mediated regulation. Among them, rs1063192 and rs3217992 have been shown previously to affect the miRNA–mRNA interaction in vitro.^21,54^ These two SNPs reside in the 3′UTR of *CDKN2B* and are not in strong LD (*R*^2^ > 0.8) with any other known *CDKN2B* variants. The *CDKN2B* gene encodes a cyclin-dependent kinase inhibitor and with its antisense (*CDKN2B-AS1*) lies in a well-known glaucoma-associated locus on Chr.9p21.^5,6^ The minor allele (G) at rs1063192 site is predicted to create a binding site for miR-323b-5p and the rs3217992 minor allele (T) is expected to disrupt the existing binding site of miR-138-2-3p within the 3′UTR of *CDKN2B*. Horswell et al.^54^ have shown that these miRNAs control both *CDKNB2* mRNA and protein levels. Moreover, they have shown the correlation of rs1063192 minor allele with lower *CDKN2B* mRNA levels (improving the miR-323b-5p–mediated regulation) and conversely, the correlation of rs3217992 minor allele with higher *CDKN2B* mRNA levels (disrupting the miR-138-3p–mediated regulation) in adipose tissue.^54^ Although the impact of these variants on the *CDKN2B* expression needs to be demonstrated in the ocular tissues, the functional consequences of both variants on the miRNA-binding sites have been confirmed by Luciferase reporter assays in cell lines.^21,54^ Thus, an allelic-specific regulation of *CDKN2B* by miR-232b-5p and miR-138-2-3p may be considered as a potential mechanism, at least in part, to explain the association between *CDKN2B* (rs1063192 and rs3217992) and POAG.

In addition to the two experimentally validated variants in the 3′UTR of *CDKN2B*, we found several other 3′UTR variants associated with POAG endophenotypes that are located in miRNA-binding sites and are one of the top variants in their loci with no nonsynonymous proxy in high LD (*R*^2^ > 0.8). These variants have the potential to be functional variants in their loci by affecting miRNA-mediated gene regulations and warrant further investigations. For example, the minor allele of rs1052990 in the 3′UTR of *CAV2*, a known gene for glaucoma, is predicted to disrupt the predicted binding site of miR-224-5p and increase the transcript levels of *CAV2*. The association of the rs1052990 minor allele with increased expression levels of *CAV2* has been reported previously.^55^ In addition, both miR-224-5p and *CAV2* are expressed in the eye.^56,57^ These data may suggest an allele-specific regulation of *CAV2* by miR-224-5p as a functional mechanism underlying the observed GWAS association.

Although we believe our results are reliable, there are certain limitations that need to be considered in interpreting of our results. First, the reported miRNA–target interactions are predicted from in silico approach and need to be validated by experimental studies. Second, we used eQTL data from various tissues, but not eye tissue, to check the association between the identified 3′UTR variants and the expression of hosting genes. As gene expression and eQTL are tissue specific, in an optimal setting one should examine the associations in the ocular tissues. Finally, we were not able to perform the functional experiments in a relevant cell line for glaucoma disease, owing to slow growth rate and low transfection efficiency of retinal ganglion cells in culture. However, to gain insights into the impact of the identified variant on miR-4707, we performed our experiments with HEK293 cells, a commonly used cell line in this type of studies.

## Conclusions

We systematically investigated the association of miRNA-related genetic variants with four POAG endophenotypes. We found variants in the miR-612 precursor and in the miR-4707 seed region that are significantly associated with VCDR and cup area. Our results showed that the minor allele of these variants may alter expression or targeting of the miRNAs. Further, we highlighted a number of 3′UTR variants associated with POAG endophenotypes that are located in the predicted miRNA-binding sites and may affect miRNA-mediated gene regulation in POAG. The identified miRNAs and target genes are candidates for future studies to determine their roles in the pathophysiology of POAG and their therapeutic potentials for glaucoma.

## Supplementary Material

Supplement 1

## Appendix

### International Glaucoma Genetics Consortium (IGGC) Membership List

Tin Aung,^1–3^ Kathryn P. Burdon,^4^ Ching-Yu Cheng,^1–3^ Jessica N. Cooke Bailey,^5^ Jamie E. Craig,^6^ Angela J. Cree,^7^ Paul J. Foster,^8^ Christopher J. Hammond,^9^ Alex W. Hewitt,^10,11^ René Höhn,^12,13^ Pirro G. Hysi,^9^ Jost Jonas,^14^ Anthony P. Khawaja,^8,15^ Andrew J. Lotery,^7^ Stuart MacGregor,^16^ David A. Mackey,^17^ Paul Mitchell,^18^ Louis R. Pasquale,^19,20^ Francesca Pasutto,^21^ Norbert Pfeiffer,^22^ Ananth C. Viswanathanm,^8^ Veronique Vitart,^23^ Eranga N. Vithana,^1^ Jie Jin Wang,^18^ Janey L. Wiggs,^19^ Robert Wojciechowski,^24–26^ Tien Yin Wong,^1–3^ and Terri L. Young^27^

^1^Singapore Eye Research Institute, Singapore National Eye Centre, Singapore.

^2^Ophthalmology & Visual Sciences Academic Clinical Program (Eye ACP), Duke-NUS Medical School, Singapore.

^3^Department of Ophthalmology, Yong Loo Lin School of Medicine, National University of Singapore, Singapore.

^4^Menzies Institute for Medical Research, University of Tasmania, Hobart, Tasmania, Australia.

^5^Department of Epidemiology and Biostatistics, Case Western Reserve University, Cleveland, Ohio, United States.

^6^Department of Ophthalmology, Flinders University, Adelaide, Australia.

^7^Clinical & Experimental Sciences, Faculty of Medicine, University of Southampton, Southampton, United Kingdom.

^8^NIHR Biomedical Research Centre, Moorfields Eye Hospital NHS Foundation Trust and UCL Institute of Ophthalmology, London, United Kingdom.

^9^Department of Twin Research and Genetic Epidemiology, King's College London, United Kingdom.

^10^Centre for Eye Research Australia, University of Melbourne, Department of Ophthalmology, Royal Victorian Eye and Ear Hospital, Melbourne, Victoria, Australia.

^11^School of Medicine, Menzies Institute for Medical Research, University of Tasmania, Hobart, Tasmania, Australia.

^12^Department of Ophthalmology, University Medical Center Mainz, Mainz, Germany.

^13^Department of Ophthalmology, Inselspital, University Hospital Bern, University of Bern, Switzerland.

^14^Department of Ophthalmology, Medical Faculty Mannheim of the Ruprecht-Karls-University of Heidelberg, Mannheim, Germany.

^15^Department of Public Health and Primary Care, Institute of Public Health, University of Cambridge School of Clinical Medicine, Cambridge, United Kingdom.

^16^Statistical Genetics, QIMR Berghofer Medical Research Institute, Brisbane, Australia.

^17^Lions Eye Institute, Centre for Ophthalmology and Visual Science, University of Western Australia, Perth, Western Australia, Australia.

^18^Centre for Vision Research, Department of Ophthalmology and Westmead Institute for Medical Research, University of Sydney, Sydney, New South Wales, Australia.

^19^Department of Ophthalmology, Harvard Medical School and Massachusetts Eye and Ear Infirmary, Boston, Massachusetts, United States.

^20^Channing Division of Network Medicine, Brigham and Women's Hospital, Boston, Massachusetts, United States.

^21^Institute of Human Genetics, Friedrich-Alexander-Universität Erlangen-Nürnberg (FAU), Erlangen, Germany.

^22^Department of Ophthalmology, University Medical Center Mainz, Mainz, Germany.

^23^Institute of Genetics and Molecular Medicine, Medical Research Council Human Genetics Unit, University of Edinburgh, Edinburgh, United Kingdom.

^24^Computational and Statistical Genomics Branch, National Human Genome Research Institute (NIH), Baltimore, Maryland, United States.

^25^Department of Epidemiology, Johns Hopkins Bloomberg School of Public Health, Baltimore, Maryland, United States.

^26^Wilmer Eye Institute, Johns Hopkins School of Medicine, Baltimore, Maryland, United States.

^27^Department of Ophthalmology and Visual Sciences, University of Wisconsin School of Medicine and Public Health, Madison, Wisconsin, United States.
