# Supplementary material for: A Genome-Wide Scan for MicroRNA-Related Genetic Variants Associated With Primary Open-Angle Glaucoma
Source: Invest Ophthalmol Vis Sci. 2017 Oct;58(12):5368–77. doi: 10.1167/iovs.17-22410 (PMC6110129; doi:10.1167/iovs.17-22410)
Supplement: Supplement 1 [file IOVS-17-22410-s01.pdf]

Supplementary data

Table S1. List of the primers for the cloning of precursor miR-4707

Table S2. Baseline characteristics of the Rotterdam Study for interaction analysis

Table S3. List of the primers for the cloning of 3'UTR sequence of *CARD10*

Table S4. List of databases and web tools used for functional annotation of miRNA-binding site SNPs associated with POAG endophenotypes

Table S5. MiRNA genetic variants nominally associated with POAG endophenotypes

Table S6. miR-612 and miR-4707-3p putative target genes associated with VCDR and cup area

Table S7. Expression of the putative miR-612 and miR-4707-3p target genes associated with VCDR and cup area across different eye tissues

Table S8. The miRNA-target gene interaction analysis using the Rotterdam Study data

Table S9. Forty-seven miRNA-binding site variants within the 3'UTRs of 21 genes associated with POAG endophenotypes

Table S10. Functional characteristics of 47 miRNA-binding site variants associated with POAG endophenotypes

Table 11. Expression of genes hosting the 47 miRNA-binding site variants associated with POAG endophenotypes across eye tissues

Table S12. Prioritization of 47 miRNA-binding site variants associated with POAG endophenotypes

Figure S1. The predicted hairpin structure of pre-miR-612 and pre-miR-4707 and the position of two identified variants associated with VCDR and cup area

Figure S2. Manhattan plots showing the association of 72,052 miRNA-binding site variants with POAG endophenotypes

Figure S3. Regional plots showing the association of ten highlighted miRNA-binding site variants with POAG endophenotypes

Table S1. List of the primers for the cloning of precursor miR-4707

Oligo name	Sequence (5'-3')
miR-4707_WT_F	5’TCGAGGGTTCCGGAGCCCCGGCGCGGGCGGGTTCTGGGGTGTAGACGCTGC TGGCCAGCCC**G**CCCCAGCCGAGGTTCTCGGCACCG 3’
miR-4707_WT_R	5’AATTCGGTGCCGAGAACCTCGGCTGGGG**C**GGGCTGGCCAGCAGCGTCTACA CCCCAGAACCCGCCCGCGCCGGGGCTCCGGAACCC 3’
miR-4707_Mut_F	5’TCGAGGGTTCCGGAGCCCCGGCGCGGGCGGGTTCTGGGGTGTAGACGCTGC TGGCCAGCCC**U**CCCCAGCCGAGGTTCTCGGCACCG 3’
miR-4707_Mut_R	5’AATTCGGTGCCGAGAACCTCGGCTGGGT**A**GGGCTGGCCAGCAGCGTCTACA CCCCAGAACCCGCCCGCGCCGGGGCTCCGGAACCC 3’

WT, Wild type pre-miRNA sequence containing allele G at the rs2273626 SNP site; Mut, Mutant pre-miRNA containing allele A at the rs2273626 SNP site; F, Forward; R, Reverse. The restriction enzymes XhoI and EcoRI (with sticky end) are shown in red and the position of SNP is underlined in the pre-miRNA sequence.

Table S2. Baseline characteristics of the Rotterdam Study for interaction analysis

	**Total**
Number of participants	10,404
Age (years)	64.4 (9.1)
Sex (male)	4441 (42.7 %)
Body mass index (kg/m^2)	27.28 (9.14)
Systolic blood pressure (mmHg)	140.83 (25.66)
Diastolic blood pressure (mmHg)	75.76 (18.46)

Data are mean (SD) or n (%)

Table S3. List of the primers for the cloning of 3’UTR of *CARD10* gene

Oligo name	Sequence (5'-3')
CARD10_F	5’CTAGATGTAAATGTCACGCCCCACCCCTGTTTCATGTGGGCACTAACACGTGTGCGTTCCT**GGCGGGCA**CACTCAGGACGGGCC 3’
CARD10 _R	5’CGTCCTGAGTGTGCCCGCCAGGAACGCACACGTGTTAGTGCCCACATGAAACAGGGGTGGGGCGTGACATTTACAT 3’

F, Forward; R, Reverse. The restriction enzymes XbaI and ApaI (sticky end) are shown in red and the binding site of miR-4707-3p within the 3’UTR of *CARD10* is underlined.

Table S4. List of databases and web tools used for functional annotation of miRNA-binding site SNPs associated with POAG endophenotypes

Name	Main feature	URL
**miRNASNP v2**	SNPs in miRNA-related sequences	http://bioinfo.life.hust.edu.cn/miRNASNP2/index.php
**PolymiRTS 3.0**	SNPs in miRNA binding sites	http://compbio.uthsc.edu/miRSNP
**Vienna RNA**	Prediction of SNP effect on miRNA secondary structure	http://rna.tbi.univie.ac.at
**TargetScan 7.0**	miRNA target prediction	http://www.targetscan.org
**miRDB**	miRNA target prediction	http://mirdb.rog /
**IPA**	A comprehensive software on biological and pathway analysis	http://www.ingenuity.com/products/ipa
**OTDB**	Gene expression data across different human eye tissues from the Ocular Tissue Database	https://genome.uiowa.edu/otdb/
**HMDD**	The human miRNA expression database	http://bioinfo.life.hust.edu.cn/smallRNA/index.php
**SNAP**	Proxy SNPs based on linkage disequilibrium and physical distance	https://www.broadinstitute.org/mpg/snap/ldsearch.php
**HaploReg v4.1**	For exploring annotations of variants on haplotype blocks	http://www.broadinstitute.org/mammals/haploreg/haploreg.php
**Genenetwork**	Whole blood eQTL data	http://www.genenetwork.org/webqtl/main.py
**GTEx**	eQTL data in other tissue	http://www.gtexportal.org/home
**miRBase**	miRNA information	http://www.mirbase.org
**GeneCards**	A database of human genes	http://www.genecards.org/
**LocusZoom**	Generating regional association plots	http://www.locuszoom.org/

Table S5. MiRNA genetic variants nominally associated with POAG endophenotypes

miRNA ID	Location in miR	SNP ID	A1	A2	Freq A1	Chr	Effect	P-value	Pheno
miR-1178	seed	rs7311975	t	c	0.9683	12	-0.0285	0.01393	DA
miR-1178	premiR	rs74614893	a	g	0.0296	12	0.0107	0.01327	VCDR
miR-1208	premiR	rs2648841	t	g	0.2379	8	0.0033	0.03801	VCDR
miR-1208	premiR	rs56863230	c	g	0.0644	8	0.3185	0.02324	IOP
miR-1258	premiR	rs146754630	t	c	0.9715	2	0.0121	0.0349	VCDR
miR-1269b	seed	rs7210937	c	g	0.2272	17	0.0066	0.04866	CA
miR-1269b	seed	rs7210937	c	g	0.2452	17	0.0036	0.03398	VCDR
miR-1283-1	premiR	rs57111412	a	g	0.8095	19	0.0103	0.009886	CA
miR-1343	premiR	rs2986407	t	c	0.2074	11	0.0101	0.02674	DA
miR-199b	premiR	rs146892675	t	c	0.013	9	-0.0478	0.01127	CA
miR-300	premiR	rs12894467	t	c	0.4661	14	0.0097	0.02933	DA
miR-3144	mature	rs67106263	a	g	0.215	6	-0.0063	0.02388	CA
miR-3144	premiR	rs68035463	a	c	0.215	6	-0.0063	0.02384	CA
miR-3152	mature	rs13299349	a	g	0.292	9	-0.0088	0.000193	CA
miR-3152	mature	rs13299349	a	g	0.2912	9	-0.0031	0.01156	VCDR
miR-3166	premiR	rs35854553	a	t	0.1006	11	-0.0869	0.04025	IOP
miR-320e	premiR	rs10423365	a	g	0.4807	19	0.0056	0.00788	CA
miR-339	mature	rs72631820	t	c	0.9883	7	0.1433	0.00315	DA
miR-339	mature	rs72631820	t	c	0.9886	7	-0.6106	0.02465	IOP
miR-3609	premiR	rs149626867	a	t	0.9899	7	-0.1531	0.04763	CA
miR-3612	premiR	rs1683709	a	g	0.291	12	-0.0088	0.04418	DA
miR-3659	premiR	rs553122	t	c	0.0271	1	-0.138	0.005187	DA
miR-3683	premiR	rs6943868	a	t	0.0413	7	0.0248	0.03751	DA
miR-3686	premiR	rs6997249	a	g	0.2902	8	0.0571	0.03669	IOP
miR-3909	premiR	rs9607265	a	g	0.0345	22	-0.0677	0.03072	DA
miR-3928	premiR	rs5997893	a	g	0.3983	22	0.0024	0.03375	VCDR
miR-3936	premiR	rs367805	t	c	0.3522	5	0.0099	0.01168	DA
miR-411	premiR	rs111906529	t	c	0.9867	14	-0.0231	0.005906	VCDR
miR-4268	premiR	rs4674470	t	c	0.7696	2	0.0091	0.04594	DA
miR-4274	premiR	rs12512664	a	g	0.6065	4	-0.0126	0.004623	DA
miR-4472-1	seed	rs28655823	c	g	0.1145	8	-0.0053	0.006561	VCDR
miR-4480	premiR	rs7912031	t	g	0.9381	10	-0.0263	0.03441	CA
miR-4480	premiR	rs7912031	t	g	0.9277	10	0.266	0.04299	IOP
miR-4482	premiR	rs641071	t	g	0.3494	10	-0.058	0.01807	IOP
miR-4513	seed	rs2168518	a	g	0.599	15	0.0663	0.008575	IOP
miR-4532	seed	rs60432575	a	g	0.0532	20	-0.0384	0.03172	DA
miR-4532	seed	rs60432575	a	g	0.0593	20	-0.0109	0.03875	VCDR
miR-4634	premiR	rs7709117	a	g	0.6109	5	-0.0025	0.04972	VCDR
miR-4655	mature	rs78996026	t	c	0.0337	7	0.8643	0.03424	IOP
miR-4707	seed	rs2273626	a	c	0.5674	14	-0.0083	9.93E-05	CA
miR-4707	seed	rs2273626	a	c	0.5817	14	-0.0046	9.45E-05	VCDR

miR-4743	premiR	rs7235219	a	g	0.984	18	-0.0262	0.04093	CA
miR-4743	premiR	rs7235219	a	g	0.9873	18	-0.0204	0.002629	VCDR
miR-4749	mature	rs147943327	t	c	0.012	19	-0.261	0.04944	DA
miR-4781	seed	rs74085143	a	g	0.0359	1	0.0132	0.04877	CA
miR-4781	seed	rs74085143	a	g	0.0365	1	0.0312	0.007822	DA
miR-4791	premiR	rs77021670	a	g	0.9894	3	-1.2754	0.01588	IOP
miR-4792	premiR	rs11714172	t	g	0.5881	3	0.0537	0.0304	IOP
miR-492	premiR	rs2289030	c	g	0.1246	12	0.1195	0.004177	IOP
miR-499b	mature	rs3746444	a	g	0.8069	20	0.0061	0.02291	CA
miR-499b	mature	rs3746444	a	g	0.8056	20	-0.0101	0.02867	DA
miR-499b	mature	rs3746444	a	g	0.8041	20	-0.0751	0.008883	IOP
miR-520f	premiR	rs75598818	a	g	0.0498	19	-0.0237	0.009902	CA
miR-548ae-2	premiR	rs10461441	a	g	0.8964	5	-0.0138	0.04161	DA
miR-548al	seed	rs515924	a	g	0.8083	11	-0.0082	0.002708	CA
miR-548al	seed	rs515924	a	g	0.8099	11	0.0857	0.004542	IOP
miR-548al	seed	rs515924	a	g	0.8063	11	-0.0031	0.02442	VCDR
miR-548ap	mature	rs4414449	a	g	0.6176	15	0.0081	0.03079	DA
miR-548ap	premiR	rs4577031	a	t	0.3825	15	-0.0082	0.02995	DA
miR-548h-4	premiR	rs184537764	a	g	0.9696	8	0.0171	0.02706	CA
miR-548h-4	premiR	rs184537764	a	g	0.9715	8	0.0087	0.03774	VCDR
miR-550a-3	premiR	rs2696977	t	c	0.9825	7	-0.0297	0.04466	VCDR
miR-550a-3	premiR	rs850108	a	g	0.763	7	0.0045	0.02953	VCDR
miR-564	premiR	rs2292181	c	g	0.0549	3	0.0116	0.01352	CA
miR-5680	premiR	rs487571	t	c	0.384	8	0.0043	0.04257	CA
miR-5682	premiR	rs9877402	a	g	0.9468	3	0.1559	0.005032	IOP
miR-5689	mature	rs9295535	t	c	0.7457	6	-0.007	0.005395	CA
miR-5689	mature	rs9295535	t	c	0.7341	6	-0.0038	0.002771	VCDR
miR-5700	mature	rs12314280	t	c	0.8986	12	0.0816	0.03086	IOP
miR-596	mature	rs61388742	t	c	0.8886	8	0.0112	0.01198	CA
miR-597	premiR	rs79397096	a	g	0.0125	8	0.0827	0.02142	DA
miR-612	premiR	rs12803915	a	g	0.2014	11	-0.0136	1.21E-06	CA
miR-612	premiR	rs12803915	a	g	0.1978	11	-0.0087	4.63E-09	VCDR
miR-6130	premiR	rs76118570	a	t	0.9879	21	-0.2875	0.000543	DA
miR-656	premiR	rs58834075	t	c	0.0264	14	-0.038	0.01921	DA
miR-656	premiR	rs58834075	t	c	0.0277	14	-0.0144	0.002196	VCDR
miR-6719	premiR	rs182936228	a	g	0.0519	19	0.0123	0.04389	VCDR
miR-6729-5p	Mature	rs75036690	a	g	0.0494	1	-0.0427	0.04069	DA
miR-7854-3p	Mature	rs2925980	a	g	0.6452	16	0.0085	0.0285	DA
miR-941-3	premiR	rs7360929	t	c	0.9589	20	0.0138	0.02685	VCDR

Shown are 63 genetic variants located in 59 unique miRNAs that are nominally ($p < 0.05$) associated with POAG endophenotypes in the publicly available IGGC GWAS data.

Table S6. The putative miR-612 and miR-4707-3p target genes associated with VCDR and cup area

miRNA ID	Phenotype	All target genes (number of SNPs)	VCDR-cup area-associated putative targets	Top SNP	GWAS p-value
miR-612	VCDR	717 (118,327)	*USH2A*	rs17025860	1.06E-120
			FAM101A	rs11057482	4.30E-09
			JRK	rs2978978	1.10E-08
			SIX4	rs17834412	6.63E-08
miR-612	Cup area	717 (118,197)	*SIX4*	rs17834412	4.0E-17
			FAM101A	rs2001506	7.01E-13
miR-4707-3p	VCDR	1159 (131,887)	*MYPN*	rs10733840	4.43E-31
			CARD10	rs6000755	3.52E-12
			LTBP3	rs12270054	5.69E-09
			H1F0	rs5756825	1.54E-08
			FLNB	rs6764184	1.89E-08
			SLC25A16	rs7906309	2.57E-08
			FRMD8	rs1626021	2.27E-07
miR-4707-3p	Cup area	1159 (131,758)	***FLNB***	rs6764184	1.30E-10
			MYPN	rs10733840	2.57E-09
			PFAS	rs4791641	3.46E-07

Shown are putative target genes of miR-612 and miR-4707-3p associated with VCDR and cup area (using the publicly available GWAS data of POAG endophenotypes). TargetScan v7.0 (total context++ score > 0.01) and miRDB databases were used to extract the predicted target genes of the miRNAs. The Bonferroni correction was used (based on the number of SNPs in the studied target genes of each miRNA) to set the significance level, which is p-value < 4.2E-07 for miR-612 target genes and p-value < 3.8E-07 for miR-4707 target genes. The SNPs shown in the table are top SNPs in the mentioned target genes associated with VCDR and cup area. Bold target genes have been shown previously to be differentially expressed in the eye tissues (trabecular meshwork, aqueous humor or optic nerve) in the previous gene expression profiling studies of glaucoma patients.

Table S7. Expression of the putative miR-612 and miR-4707-3p target genes associated with VCDR and cup area across different eye tissues

Gene	Probe ID	Choroid RPE	Ciliary Body	Cornea	Optic Nerve	Retina	Sclera	Trabecular Meshwork
USH2A	2455699	22.6	21.3	15.9	20.1	237.9	19.9	16.8
FAM101A	3436329	32.8	37.7	28.5	30.6	28.9	39.7	29.5
JRK	3157060	44.5	40.7	52.8	50.4	35.6	50.4	43.7
SIX4	3567391	83.9	81.3	118.4	121.2	86.5	110.9	86.0
MYPN	3249641	22.2	20.0	21.1	18.2	22.3	19.3	16.7
CARD10	3960133	60.0	60.3	68.6	67.0	70.0	83.8	66.6
LTBP3	3377669	69.9	60.8	75.0	76.4	60.1	88.1	73.9
H1F0	3945006	118.3	103.2	85.5	84.9	88.2	125.9	171.0
FLNB	2625907	68.3	67.8	74.5	87.1	36.8	100.7	123.5
PFAS	3709540	34.1	35.1	35.1	32.5	40.2	39.8	30.3
SLC25A16	3292735	60.8	38.8	59.1	48.7	70.4	53.4	75.5
FRMD8	4010913	28.8	31.4	62.8	72.1	72.8	36.4	45.4

Shown are expression of a number of miR-612 and miR-4707-3p predicted target genes, which found to be significantly associated with VCDR and cup area, on eye tissues using the online Ocular Tissue Database. The gene expression is indicated as Affymetrix Probe Logarithmic Intensity Error (PLIER) number. The PLIER number were calculated by GC-background correction, PLIER normalization, log transformation and z-score calculation.

Table S8. The miRNA-target gene interaction analysis using the Rotterdam Study data

miRNA	SNP ID	Target gene	Top SNP	Cup area (*p*-value)	VCDR (*p*-value)
miR-612	rs12803915	*SIX4*	rs17834412	0.12	0.19
		USH2A	rs17025860	-	-
		FAM101A	rs11057482	-	-
		JRK	rs2978978	-	-
miR-4707	rs2273626	*MYPN*	rs10733840	0.97	0.38
		CARD10	**rs6000755**	**0.028**	0.06
		LTBP3	rs4244811	0.9	0.95
		H1F0	rs5756825	0.92	0.89
		FRMD8	rs1626021	0.82	0.91
		FLNB	rs6764184	-	-
		SLC25A16	rs7906309	-	-
		PFAS	rs4791641	-	-

This table shows the interaction analysis between two identified SNPs in miR-612 and miR-4707 with the top SNP in their target genes associated with glaucoma using the Rotterdam Study (n=10,404). VCDR, the mean of vertical cup to disc ratio which is between 0 and 1 (Mean= 0.44, SD= 0.18); Cup area is between 0 and 2 (Mean= 0.54, SD= 0.34). This analysis indicates a significant interaction between miR-4707 (rs2273626) and *CARD10* (rs6000755) in relation to cup area, shown in bold.

Table S9. Forty-seven miRNA-binding site variants within the 3'UTRs of 21 genes associated with POAG endophenotypes

SNP ID	Gene ID	Chr.	Position	Asso. pheno	MA effect	*p*-value GWAS	A1	miRNA binding sites in presence of A1	A2	miRNA binding sites in presence of A2
rs12125947	*CDC7*	1	91990487	DA	*C (-)*	4.00E-08	t	miR-6768-3p	c	miR-4729, miR-4778-3p miR-5696, miR-579-3p miR-664b-3p
rs6660601	*TMCO1*	1	165695855	IOP	*C (+)*	4.23E-11	c	miR-296-3p, miR-323b-5p miR-410-5p, miR-494-5p miR-511-5p	t	miR-3158-3p, miR-4446-3p miR-4675, miR-4741 miR-5088-5p, miR-626 miR-6876-3p
rs7524755	*TMCO1*	1	165694897	IOP	*T (-)*	4.35E-11	c	miR-4454, miR-598-5p	t	miR-4299, miR-4456 miR-548q, miR-7978
rs1057001	*TRIB2*	2	12881778	CA	*T (+)*	1.13E-09	t	miR-545-3p, miR-583	a	miR-3125, miR-3916 miR-4310, miR-6859-5p miR-7157-5p, miR-877-5p
rs7344	*SRSF3*	6	36570366	CA	*C (+)*	3.95E-07	t	hsa-let-7f-2-3p, miR-1185-1-3p miR-1185-2-3p, miR-3120-3p	c	hsa-let-7e-3p, miR-4445-3p **miR-548al**
rs10249656	*CAV2*	7	116147537	IOP	*T(+)*	1.22E-10	c	miR-3187-3p , miR-4435 miR-4701-5p, miR-588 **miR-4781-3p**, miR-548s	t	miR-4662a-5p, miR-575 miR-4676-5p, miR-6858
rs10278782	*CAV2*	7	116147401	IOP	*G (+)*	1.54E-10	a	miR-216b-3p	g	miR-1228-3p
rs1052990	*CAV2*	7	116148370	IOP	*G (+)*	1.90E-09	g	miR-224-5p, miR-377-3p	t	-
rs10216533	*PSCA*	8	143763690	VCDR	*A (-)*	2.82E-08	a	miR-1273h-3p, **miR-3166** miR-342-5p, miR-4664-5p miR-4771, miR-6782-5p	g	miR-6767-5p
rs1045547	*PSCA*	8	143763757	VCDR	*G (+)*	2.67E-08	t	miR-6826-5p	g	**miR-597-5p**, miR-668-3p
rs1045574	*PSCA*	8	143763958	VCDR	*A (-)*	2.50E-08	g	miR-4655-5p	a	miR-296-5p, miR-6724-5p miR-6773-5p
rs1045605	*PSCA*	8	143764101	VCDR	*G (-)*	2.66E-08	c	miR-2467-5p, miR-3188 miR-3975, miR-4649-3p miR-4663, miR-485-5p miR-6884-5p, miR-8077, miR-7160	g	miR-3155a, miR-3155b miR-3934-3p, miR-484
rs2976393	*PSCA*	8	143763618	VCDR	*G (-)*	2.38E-08	c	miR-3650, miR-4455 miR-6818-5p, miR-6867-5p	g	miR-4677-3p, miR-4679 miR-6753-3p, miR-7107-3p
rs2976394	*PSCA*	8	143763622	VCDR	*T (-)*	2.53E-08	c	miR-4455, miR-6781-5p miR-6877-5p	t	miR-3934-5p, miR-4640-5p miR-4726-5p, miR-6508-3p
rs2976395	*PSCA*	8	143763750	VCDR	*A (-)*	2.58E-08	g	miR-4537	a	miR-3160-3p

rs2976396	*PSCA*	8	143764001	VCDR	*A (-)*	2.49E-08	g	miR-4421, miR-5699-3p miR-6748-3p	a	miR-2355-3p,miR-3655 miR-4431, miR-676-3p
rs8176751	*ABO*	9	136131022	IOP	*T (+)*	1.05E-08	t	miR-3692-5p, miR-5580-5p miR-6762, miR-6868-5p, miR-885-3p	c	-
rs1063192	*CDKN2B*	9	22003367	VCDR, CA	*G (-)*	5.31E-33	a	miR-382-5p	g	miR-3074-3p, miR-323b-5p miR-410-5p miR-494-5p
rs3217992	*CDKN2B*	9	22003223	CA, VCDR	*T (+)*	4.07E-18	c	miR-138-2-3p, miR-205-3p	t	miR-374c-5p, miR-655-3p
rs1162756	*HNRNPH3*	10	70102936	DA	*T (-)*	5.77E-08	a	miR-489-3p	t	-
rs3199937	*HNRNPH3*	10	70102749	DA, VCDR	*A (-)*	1.35E-24	a	miR-2115-3p, miR-361-5p	c	miR-383-5p, miR-6510-5p
rs2278675	*MYPN*	10	69971652	DA	*G (+)*	2.52E-15	c	miR-1271-5p miR-636, miR-96-5p	g	-
rs876489	*MYPN*	10	69970895	DA	*C (+)*	8.41E-09	c	miR-4761-5p, miR-624-3p	g	miR-133a-5p,miR-320e miR-4490
rs881975	*MYPN*	10	69971585	DA	*A (+)*	1.20E-08	a	**miR-339-5p**, hsa-let-7a-2-3p, hsa-let-7g-3p, miR-486-5p miR-6856-3p	g	miR-6752-3p
rs881976	*MYPN*	10	69971567	DA	*T (+)*	2.67E-15	c	miR-5008-3p, miR-548g-3p miR-6737-3p, miR-7157-3p	t	miR-4728-3p
rs1807347	*PBLD*	10	68283964	DA, VCDR	*A (-)*	3.13E-84	a	miR-133a-5p, miR-4774-5p	g	miR-138-5p, miR-7978
rs2305083	*PBLD*	10	70044937	DA, VCDR	*A (-)*	1.29E-83	a	miR-1252-5p, miR-1265	c	miR-4493, miR-4687-3p miR-605-3p, miR-6126
rs731063	*PBLD*	10	70043190	DA, VCDR	*T (-)*	4.14E-84	t	miR-3143, miR-543	c	miR-141-3p, miR-200a-3p
rs10998098	*RUFY2*	10	68378024	DA, VCDR	*A (-)*	8.54E-20	g	miR-651-3p	a	miR-3613-3p
rs1177364	*RUFY2*	10	70104799	DA	*A (-)*	1.18E-07	a	-	g	miR-32-3p, miR-3591-5p miR-3606-5p, miR-921
rs17297439	*RUFY2*	10	70103461	DA, VCDR	*A (-)*	4.26E-25	a	miR-4719	g	-
rs2281697	*RUFY2*	10	70103958	DA, VCDR	*G (-)*	4.64E-25	a	miR-6074	g	**miR-548ap/h**, miR-559 miR-8054
rs2281698	*RUFY2*	10	70104320	DA, VCDR	*C (-)*	6.82E-25	t	miR-4474-3p	c	miR-132-5p
rs3781567	*RUFY2*	10	70105178	DA, VCDR	*T (-)*	9.99E-25	t	miR-219b-3p, miR-3123 miR-6511b-5p, miR-6811-5p	c	miR-1205, miR-146a-5p miR-146b-5p, miR-3925-5p

rs3781568	*RUFY2*	10	70105286	DA, VCDR	*A (-)*	1.66E-24	a	miR-1298-3p, miR-3126-3p	c	miR-4418, miR-509-3-5p<br>miR-509-5p, miR-7153-5p<br>miR-3191-5p<br>miR-326, miR-330-5p<br>miR-518c-5p, miR-6764-3p<br>miR-6817-3p, miR-6824-3p<br>miR-6828-3p, miR-767-3p
rs1136645	*SLC25A16*	10	70242513	DA, VCDR	*C (-)*	5.37E-19	c	miR-3622b-5p, miR-4720-5p<br>miR-4799-3p, miR-5588-5p	t	**miR-564**
rs4350264	*SLC25A16*	10	70242946	DA	*A (-)*	1.35E-15	g	miR-518	a	miR-4422, miR-6835-3p
rs1044269	*CELF1*	11	47487740	IOP	*G (+)*	2.32E-07	a	miR-4721	g	miR-1273c
rs595139	*FOLH1*	11	49168300	IOP	*A (+)*	1.29E-08	g	miR-186-5p, miR-3133<br>miR-4509, miR-4744	a	miR-4668-3p, miR-548c-3p
rs948577	*MAP3K11*	11	65365318	VCDR	*C (-)*	2.47E-07	c	miR-124-3p, miR-1295b<br>miR-1912, miR-500b-3p, miR-506-3p	t	miR-3910, miR-520a-5p<br>miR-525-5p
rs12421026	*PAX6*	11	31809322	DA	*T (+)*	5.53E-08	t	miR-1287-5p, miR-3135b<br>miR-6081	c	miR-1203
rs1506	*PAX6*	11	31810298	DA	*T (-)*	6.34E-09	a	miR-1537-5p, miR-3620-3p<br>miR-370-3p, miR-4718<br>miR-4999-5p, miR-6893-3p	t	miR-500b-3p
rs3026401	*PAX6*	11	31807524	DA	*C (-)*	4.21E-09	t	miR-5681b, miR-5696<br>miR-579-3p, miR-664a-3p<br>miR-664b-3p	c	miR-5579-5p
rs1043207	*RBM23*	14	23369994	VCDR	*A (+)*	6.53E-07	g	miR-3924	a	miR-190a-3p, miR-5011-5p<br>miR-6083
rs1050285	*AAGAB*	15	67494494	IOP	*C (-)*	2.89E-07	t	miR-329-5p, miR-4659a-3p<br>miR-4659b-3p, miR-6875-3p	c	miR-3667-3p, miR-6734-3p<br>miR-6868-3p
rs1132554	*PFAS*	17	8173767	CA	*T (-)*	5.53E-07	c	miR-149-3p, miR-3184-5p<br>miR-423-5p, miR-4489<br>miR-4728-5p, miR-6085<br>miR-6785-5p, miR-6813-5p<br>miR-6825-5p, miR-6883-5p	t	miR-3175, miR-3975<br>miR-6731-5p, miR-8085
rs3171656	*TRIOBP*	22	38172213	VCDR	*A (+)*	3.16E-07	g	miR-3663-3p	a	miR-30a-3p, miR-30d-3p<br>miR-30e-3p, miR-5003-3p

Shown are 47 glaucoma-associated variants that are predicted to affect miRNA-binding sites using PolymiRTS (v3.0). DA, disc area; CA, cup area; Chr, chromosome; MA, minor allele; A1, ancestral allele; A2, alternative allele. Bold miRNAs have been shown previously to be differentially expressed in the eye tissues (trabecular meshwork, aqueous humor or optic nerve) in the previous miRNA expression profiling studies of glaucoma patients.

Table S10. Functional characteristics of 47 miRNA-binding site variants associated with POAG endophenotypes

SNP ID	Host gene	A1	A2	Chr.	Position	MAF (Europe)	Proxy SNPs (R^2 > 0.8)	Non-syn. Proxies	cis-eQTL (tissue)	Effect allele in eQTL (β)
**rs12125947**	*CDC7*	t	c	1	91990487	0.49	15	0	Brain, Nerve, Blood, other tissues	*C (-)*
**rs6660601**	*TMCO1*	c	t	1	165695855	0.15	33	0	Nerve, Blood, Artery, other tissues	*C (-)*
rs7524755	*TMCO1*	c	t	1	165694897	0.15	33	0	Nerve, Blood, Artery, other tissues	*T (-)*
**rs1057001**	*TRIB2*	t	a	2	12881778	0.49	10	0	Nerve, Whole blood	*T (-)*
**rs7344**	*SRSF3*	t	c	6	36570366	0.25	64	0	Nerve, Whole blood, Skin, Thyroid	*C (+)*
**rs10249656**	*CAV2*	c	t	7	116147537	0.15	10	1	Pancreases, Testis	*T (-)*
rs10278782	*CAV2*	a	g	7	116147401	0.15	10	1	Pancreases, Blood	*G (-)*
**rs1052990**	*CAV2*	g	t	7	116148370	0.34	11	0	Pancreases, Blood, Lung, Testis	*G (-)*
**rs10216533**	*PSCA*	a	g	8	143763690	0.44	48	0	Brain, Nerve, Blood, other tissues	*A (+)*
rs1045547	*PSCA*	t	g	8	143763757	0.44	48	0	Brain, Nerve, Blood, other tissues	*G (+)*
rs1045574	*PSCA*	g	a	8	143763958	0.44	48	0	Brain, Nerve, Blood, other tissues	*A (+)*
rs1045605	*PSCA*	c	g	8	143764101	0.44	48	0	Brain, Nerve, Blood, other tissues	*G (+)*
rs2976393	*PSCA*	c	g	8	143763618	0.45	48	0	Brain, Nerve, Blood, other tissues	*G (+)*
rs2976394	*PSCA*	c	t	8	143763622	0.44	48	0	Brain, Nerve, Blood, other tissues	*T (+)*
rs2976395	*PSCA*	g	a	8	143763750	0.44	48	0	Brain, Nerve, Blood, other tissues	*A (+)*
rs2976396	*PSCA*	g	a	8	143764001	0.44	48	0	Brain, Nerve, Blood, other tissues	*A (+)*
**rs8176751**	*ABO*	t	c	9	136131022	0.11	7	1	Nerve, Adipose, Heart, Lung, Skin	*T (+)*
**rs1063192**	*CDKN2B*	a	g	9	22003367	0.43	29	0	Temporal cortex	*G (-)*
**rs3217992**	*CDKN2B*	c	t	9	22003223	0.40	4	0	Whole blood	*T (+)*
**rs2278675**	*MYPN*	c	g	10	69971652	0.46	11	0	Muscle skeletal	*G (+)*
rs881976	*MYPN*	c	t	10	69971567	0.46	11	0	Muscle skeletal	*T (+)*
**rs876489**	*MYPN*	c	g	10	69970895	0.35	22	0	-	*C (+)*
rs881975	*MYPN*	a	g	10	69971585	0.35	22	0	-	*A (+)*

**rs1807347**	*PBLD*	a	g	10	68283964	0.20	30	1	Brain, Nerve, Liver, other tissues	*A (+)*
rs2305083	*PBLD*	a	c	10	70044937	0.20	30	1	Brain, Nerve, Liver, other tissues	*A (+)*
rs731063	*PBLD*	t	c	10	70043190	0.20	30	1	Brain, Nerve, Liver, other tissues	*T (+)*
**rs1162756**	*HNRNPH3*	a	t	10	70102936	0.03	30	1	-	*T (-)*
rs1177364	*RUFY2*	a	g	10	70104799	0.03	30	1	-	*A (-)*
**rs17297439**	*RUFY2*	a	g	10	70103461	0.11	81	0	Brain, Nerve, Blood, other tissues	*A (+)*
rs2281697	*RUFY2*	a	g	10	70103958	0.11	81	0	Brain, Nerve, Blood, other tissues	*G (+)*
rs2281698	*RUFY2*	t	c	10	70104320	0.11	81	0	Brain, Nerve, Blood, other tissues	*C (+)*
rs3781567	*RUFY2*	t	c	10	70105178	0.12	81	0	Brain, Nerve, Blood, other tissues	*T (+)*
rs3781568	*RUFY2*	a	c	10	70242946	0.12	81	0	Brain, Nerve, Blood, other tissues	*A (+)*
rs10998098	*RUFY2*	g	a	10	68378024	0.11	81	0	Brain, Nerve, Blood, other tissues	*A (+)*
rs3199937	*HNRNPH3*	a	c	10	70102749	0.11	81	0	Brain, Nerve, Blood, other tissues	*A (-)*
**rs1136645**	*SLC25A16*	c	t	10	70242513	0.13	26	0	Brain, Nerve, Blood, other tissues	*C (-)*
**rs4350264**	*SLC25A16*	g	a	10	70105286	0.11	60	0	Brain, Nerve, Blood, other tissues	*A (-)*
**rs1044269**	*CELF1*	a	g	11	47487740	0.15	30	0	Nerve, Muscle, Blood, other tissues	*G (+)*
**rs595139**	*FOLH1*	g	a	11	49168300	0.14	60	2	Skin, Testis	*A (+)*
**rs948577**	*MAP3K11*	c	t	11	65365318	0.27	20	0	Artery, Adipose, Blood, Skin	*C (-)*
**rs1506**	*PAX6*	a	t	11	31810298	0.17	4	0	-	*T (-)*
rs3026401	*PAX6*	t	c	11	31807524	0.19	4	0	-	*C (-)*
**rs12421026**	*PAX6*	t	c	11	31809322	0.49	3	0	Lymphoblastoid	*T (-)*
**rs1043207**	*RBM23*	g	a	14	23369994	0.26	1	0	Brain, Nerve, Blood, other tissues	*A (-)*
**rs1050285**	*AAGAB*	t	c	15	67494494	0.48	74	0	Adipose, Lung, Skin, Thyroid	*C (-)*
**rs1132554**	*PFAS*	c	t	17	8173767	0.45	5	0	Nerve, Blood, Adipose, Colon	*T (-)*
**rs3171656**	*TRIOBP*	g	a	22	38172213	0.34	61	2	Nerve, Blood, Adipose, other tissues	*A (-)*

The high LD proxies and eQTL of 47 miRNA-binding site SNPs associated with POAG endophenotypes using HaploReg v4.1. In total, 723 intronic and 10 missense SNPs are in LD ($R^2 > 0.8$) with the binding site SNPs. Bold are the most significant SNPs among the correlated SNPs in one gene.

Table 11. Expression of genes hosting the 47 miRNA-binding site variants associated with POAG endophenotypes in eye tissues

Gene ID	Probe ID	Trabecular Meshwork	Optic Nerve	Choroid RPE	Ciliary Body	Cornea	Lens	Retina	Sclera
AAGAB	3630450	147.6	37.1	50.1	48.7	82.2	76.4	48.7	39.1
ABO	3228582	33.3	25.7	36.3	20.2	25.6	30.9	23.3	33.6
CAV2	3020273	39.8	19.5	24.8	31.3	30.3	21	20.4	23.7
CDC7	2346399	31.4	13.3	19.4	31.1	26.3	11.0	42.1	14.1
CDKN2B	3201488	21.4	42.8	28.8	16.5	34.2	43.0	35.8	36.5
CELF1	3372253	88.4	60.2	44.1	36.6	72.2	548.2	61.1	43.2
FOLH1	3372896	28.8	164.6	53.1	47.9	24.0	37.2	22.6	32.2
HNRNPH3	3249738	646.0	699.6	736.9	619.8	568.4	983.0	704.1	523.7
MAP3K11	3377752	34.5	34.8	30.7	27.2	43.3	31.5	37.1	39.6
MYPN	3249641	16.7	18.2	22.2	20.0	21.1	26.1	22.3	19.3
PAX6	3368054	26.9	49.5	146.5	424.1	1207.9	583.4	299.4	22.4
PBLD	3292590	60.5	88.8	88.8	40.5	67.9	93.0	69.4	25.1
PFAS	3709540	30.3	32.5	34.1	35.0	35.1	35.8	40.2	39.8
PSCA	3119200	28.7	32.3	35.4	32.3	22.1	36.4	31.8	23.9
RBM23	3556888	50.5	39.4	46.2	42.0	52.4	40.8	56.3	36.9
RUFY2	3292634	108.8	166.0	109.8	90.1	92.3	139.2	118.5	74.9
SRSF3	2905118	356.1	206.2	558.1	462.2	271.3	257.9	224.6	178.9
SLC25A16	3292735	75.5	48.7	60.9	38.8	59.0	78.5	70.4	53.4
TMCO1	2442134	188.4	143.4	205.4	138.5	224.6	149.5	145.4	119.1
TRIB2	2470165	121.3	94.7	150.1	80.6	31.3	41.4	227.3	93.9
TRIOBP	3944922	30.8	28.0	35.2	29.5	37.7	36.9	17.8	28.1

Expression of 21 genes hosting the 47 miRNA-binding sites associated with glaucoma endophenotypes across human eye tissues (using The Ocular Tissue Database). The gene expression is indicated as Affymetrix Probe Logarithmic Intensity Error (PLIER) number. The PLIER number were calculated by GC-background correction, PLIER normalization, log transformation and z-score calculation.

Table S12. Prioritization of 47 miRNA-binding site variants associated with glaucoma endophenotypes

SNP ID	Top in GWAS	eQTL	MA (effect)	Proxy SNPs (# non-syn)	Gene ID	Gene exp in the eye	Gene asso with dis	MiRNA-binding site (disruption/creation)	miR exp in the eye	miR asso with dis	Score
rs3217992	✓	✓	*T (+)*	4 (0)	*CDKN2B*	✓	✓	miR-138-2-3p	✓	✓	9
rs1063192	✓	✓	*G (-)*	29 (0)	*CDKN2B*	✓	✓	miR-323-5p	✓	✓	8
rs1052990	✓	✓✓	*G (-)*	11 (0)	*CAV2*	✓	✓	miR-224-5p	✓	✓	8
rs1050285	✓	✓	*C (-)*	74 (0)	*AAGAB*	✓	✓	miR-329-5p	✓	-	8
rs10216533	✓	✓✓	*A (+)*	48 (0)	*PSCA*	✓	✓	miR-342-5p, miR-1273-3p	✓	-	8
rs1132554	✓	✓	*T (-)*	5 (0)	*PFAS*	✓	✓	miR-149-3p, miR-423-5p	✓	-	8
rs1057001	✓	✓✓	*T (-)*	10 (0)	*TRIB2*	✓	✓	miR-877-5p	✓	-	8
rs1043207	✓	✓✓	*A (-)*	1 (0)	*RBM23*	✓	✓	miR-190a-3p	✓	-	8
rs8176751	✓	✓✓	*T (+)*	7 (1)	*ABO*	✓	✓	miR-885-3p	✓	-	8
rs7344	✓	✓✓	*C (+)*	64 (0)	*SFRS3*	✓	✓	**miR-548al**, Let-7f-2-3p	✓	-	8
rs1045547	✓	✓✓	*G (+)*	48 (0)	*PSCA*	✓	✓	**miR-597-5p**	✓	-	8
rs2281697	-	✓✓	*G (+)*	81 (0)	*RUFY2*	✓	✓	**miR-548ap/h**, miR-559	✓	-	7
rs3781568	-	✓✓	*A (+)*	81 (0)	*RUFY2*	✓	✓	miR-1298-3p	✓	-	7
rs2281698	-	✓✓	*C (+)*	81 (0)	*RUFY2*	✓	✓	miR-132-5p	✓	-	7
rs10998098	-	✓✓	*A (+)*	81 (0)	*RUFY2*	✓	✓	miR-3613-3p	✓	-	7
rs3781567	-	✓✓	*T (+)*	81 (0)	*RUFY2*	✓	✓	miR-219b-3p	✓	-	7
rs10249656	✓	✓✓	*T (-)*	10 (1)	*CAV2*	✓	✓	**miR-4781-3p**, miR-575	✓	-	7
rs6660601	✓	✓✓	*C (-)*	33 (0)	*TMCO1*	✓	✓	miR-323-5p, miR-410-5p	✓	-	7
rs1045605	✓	✓✓	*G (+)*	48 (0)	*PSCA*	✓	✓	miR-485-5p	✓	-	7
rs2278675	-	✓✓	*G (+)*	11 (0)	*MYPN*	✓	✓	miR-1271-5p, miR-96-5p	✓	-	6
rs2976396	✓	✓✓	*A (+)*	48 (0)	*PSCA*	✓	✓	miR-676-3p	✓	-	6
rs2305083	✓	✓✓	*A (+)*	30 (1)	*PBLD*	✓	✓	miR-1252-5p	-	-	6
rs881976	-	✓✓	*T (+)*	11 (0)	*MYPN*	✓	✓	miR-548g-3p	✓	-	6
rs1045574	✓	✓✓	*A (+)*	48 (0)	*PSCA*	✓	✓	miR-296-5p	✓	-	6
rs10278782	✓	✓✓	*G (-)*	10 (1)	*CAV2*	✓	✓	miR-1228-3p	✓	-	6
rs12125947	-	✓✓	*C (-)*	15 (0)	*CDC7*	✓	✓	**miR-579-3p**	-	-	6
rs876489	✓	-	*C (+)*	22 (0)	*MYPN*	✓	✓	**miR-320e**, miR-133a	✓	-	6

rs1044269	-	✓	*G (+)*	30 (0)	*CELF1*	✓	✓	miR-1273c	-	-	5
rs3026401	✓	-	*C (-)*	4 (0)	*PAX6*	✓	✓	miR-664a-3p	✓	-	5
rs1506	✓	-	*T (-)*	4 (0)	*PAX6*	✓	✓	miR-370-3p	✓	-	5
rs2976393	✓	✓✓	*G (+)*	48 (0)	*PSCA*	✓	✓	miR-4677-3p	-	-	5
rs948577	-	✓✓	*C (-)*	20 (0)	*MAP3K11*	✓	✓	miR-520a, miR-525	✓	-	5
rs7524755	✓	✓✓	*T (-)*	33 (0)	*TMCO1*	✓	✓	miR-548q	✓	-	5
rs731063	-	✓✓	*T (+)*	30 (1)	*PBLD*	✓	✓	miR-543	✓	-	5
rs595139	-	✓✓	*A (+)*	60 (2)	*FOLH1*	✓	✓	miR-186-5p	✓	-	5
rs3171656	-	✓✓	*A (-)*	61 (2)	*TRIOBP*	✓	✓	miR-30a-3p	✓	-	5
rs1807347	-	✓✓	*A (+)*	30 (1)	*PBLD*	✓	✓	miR-133a-5p	✓	-	5
rs17297439	-	✓✓	*A (+)*	81 (0)	*RUFY2*	✓	✓	miR-4719	-	-	5
rs12421026	-	✓✓	*T (-)*	3 (0)	*PAX6*	✓	✓	miR-3135b	-	-	5
rs1136645	-	✓	*C (-)*	26 (0)	*SLC25A16*	✓	✓	**miR-564**	-	-	5
rs881975	-	-	*A (+)*	22 (0)	*MYPN*	✓	✓	**miR-339-5p**, Let-7a-2-3p	✓	-	5
rs3199937	-	-	*A (-)*	81 (0)	*HNRNPH3*	✓	✓	miR-361-5p	✓	-	4
rs4350264	✓	✓	*A (-)*	60 (0)	*SLC25A16*	✓	✓	miR-4422	-	-	4
rs2976395	✓	✓✓	*A (+)*	48 (0)	*PSCA*	✓	✓	miR-3160-3p	-	-	4
rs2976394	✓	✓✓	*T (+)*	48 (0)	*PSCA*	✓	✓	miR-3934-5p	-	-	4
rs1177364	-	-	*A (-)*	30 (1)	*RUFY2*	✓	✓	miR-32-3p	✓	-	3
rs1162756	-	-	*T (-)*	30 (1)	*HNRNPH3*	✓	✓	miR-489-3p	✓	-	3

Prioritization of the 47 miRNA-binding site SNPs associated with POAG endophenotypes. A functional score (between 1 and 10) was calculated for each SNP by combining the results of our predefined criteria including the strength of association, LD pattern, eQTL data, expression and importance of the related miRNA and target gene in the eye.

1. The strength of the association in GWAS results: Lead SNP ✓✓(2), One of the top 10% SNPs in the locus ✓(1), Only passed the threshold (0).
2. The eQTL data: Correlation between SNP and expression of the host gene ✓✓(2), Correlation of SNPs with expression of the nearby genes in the locus ✓(1), No correlation (0).
3. The LD pattern: No or just a few synonymous proxies (2), Several synonymous proxies, but no non-synonymous (1), Several proxies including non-synonymous proxy SNPs (0).
4. Host gene: Expressed in the eye and previously reported to be involved in POAG ✓✓(2), Expressed in the eye or involved in POAG ✓(1), Not expressed in the eye and no evidence to be involved in POAG (0).
5. miRNA: Expressed in the eye and previously reported to be involved in the eye disease ✓✓(2), Expressed in the eye or involved in the eye disease ✓(1), Not expressed in the eye and no evidence to be involved in the eye disease (0). One extra point for the miRNA-target interactions that are validated.

Figure S1. The predicted hairpin structure of pre-miR-612 and pre-miR-4707 and the position of two identified variants associated with VCDR and cup area

pre-miR-612

```
   ------   -ca     --a  c  cu   c          -        -a   a
5'       ucc   ucugg   cc ug  ggg agggcuucug agcuccuu  gc c
         |||   |||||   || ||  ||| |||||||||| ||||||||  ||
3'       agg   ggacc   gg ac  ccc ucccggggac ucggggag  cg u
   acucuc   aca     gac  u  cu   -          c        ga   a
                                                      ↑
                                                rs12803915
                                                  (G/A)
```

pre-miR-4707

```
      u   -     c     gc          -- ug -    u
5' ggu ccg gagcc cggc  gggcggguu  c  g ggug a
   ||| ||| ||||| ||||  |||||||||  |  | ||||
3' cca ggc cuugg gccg  cccgcccga  g  c ucgc g
      c   u     a     ac          cc gu g    a
                         ↑
                     rs2273626
                       (T/G)
```

Figure S2. Manhattan plots showing the association of 72,052 miRNA-binding site variants with POAG endophenotypes

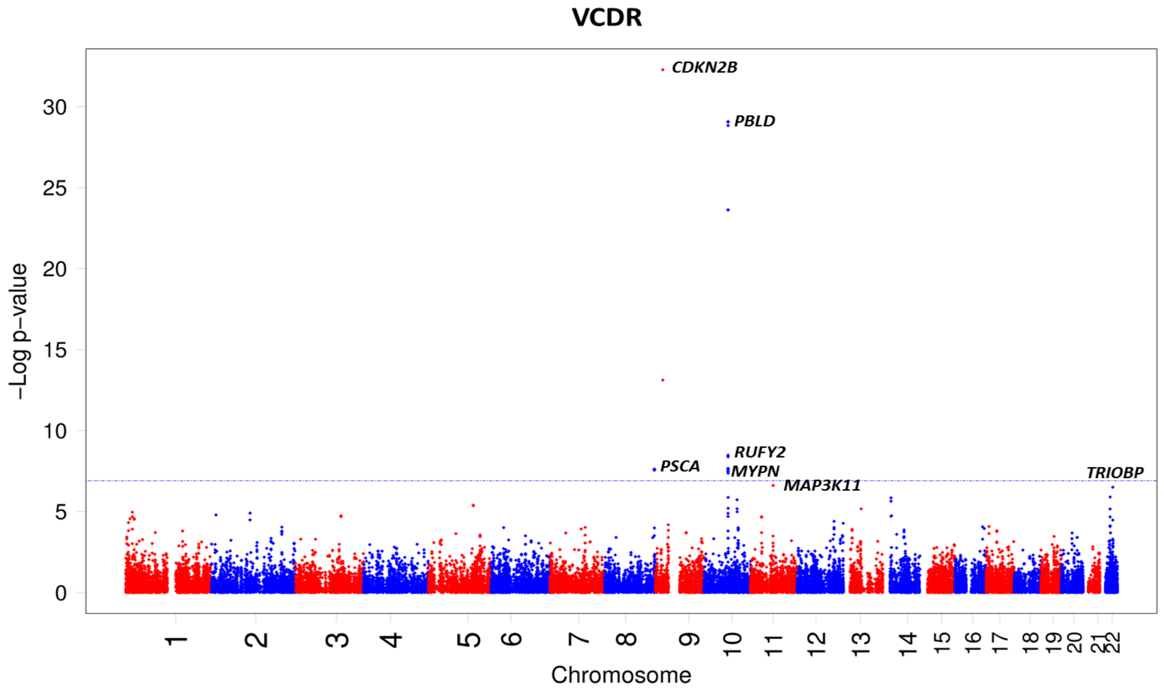

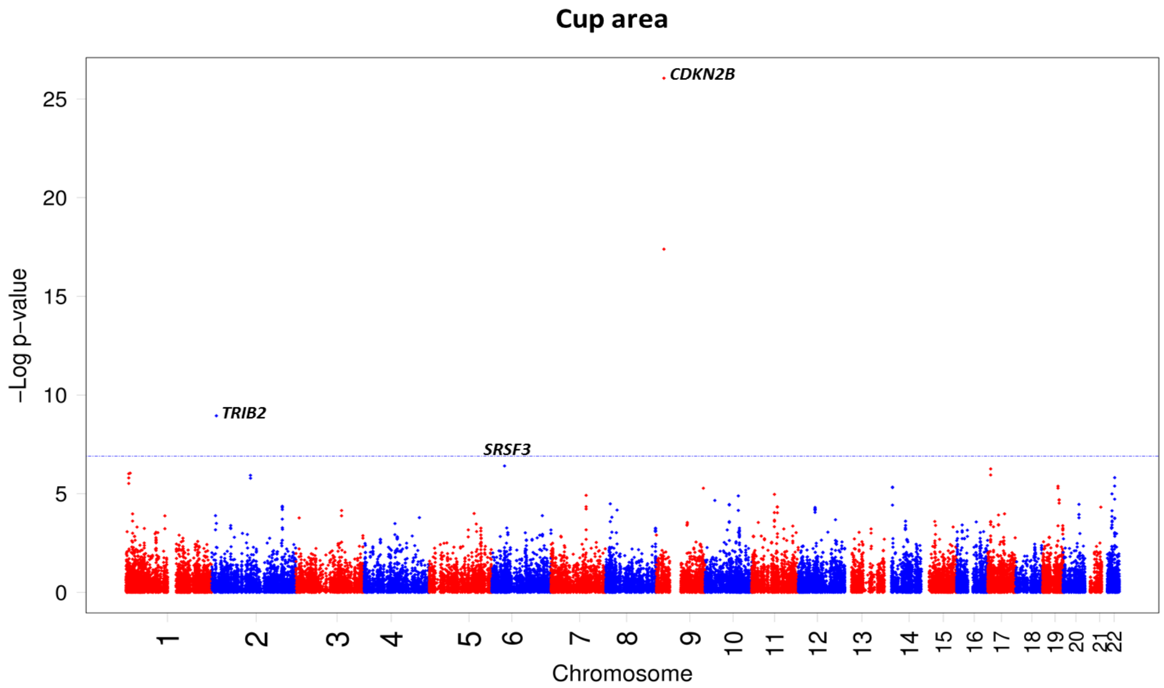

IOP

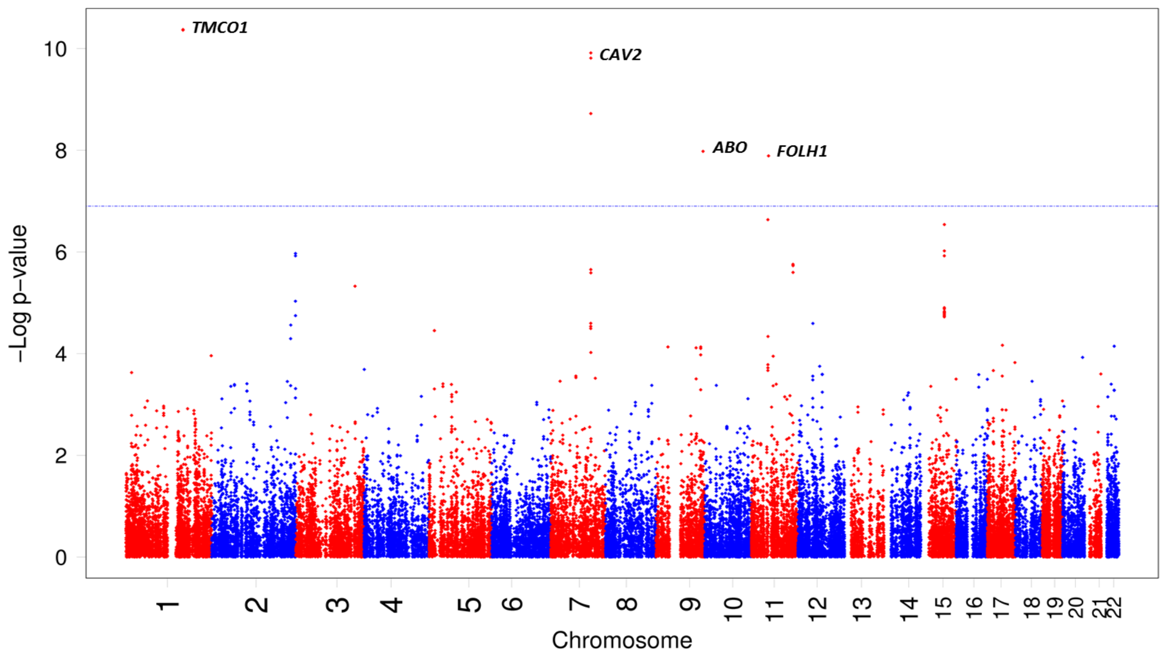

Disc area

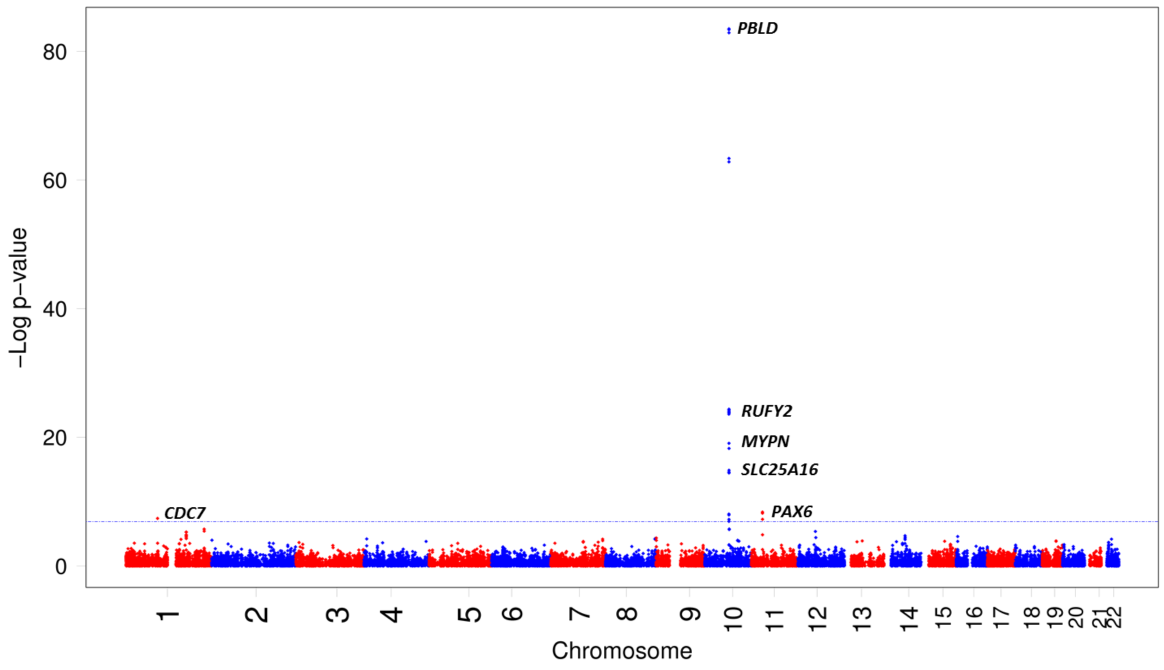

Figure S3. Regional plots showing the association of ten highlighted miRNA-binding site variants with POAG endophenotypes

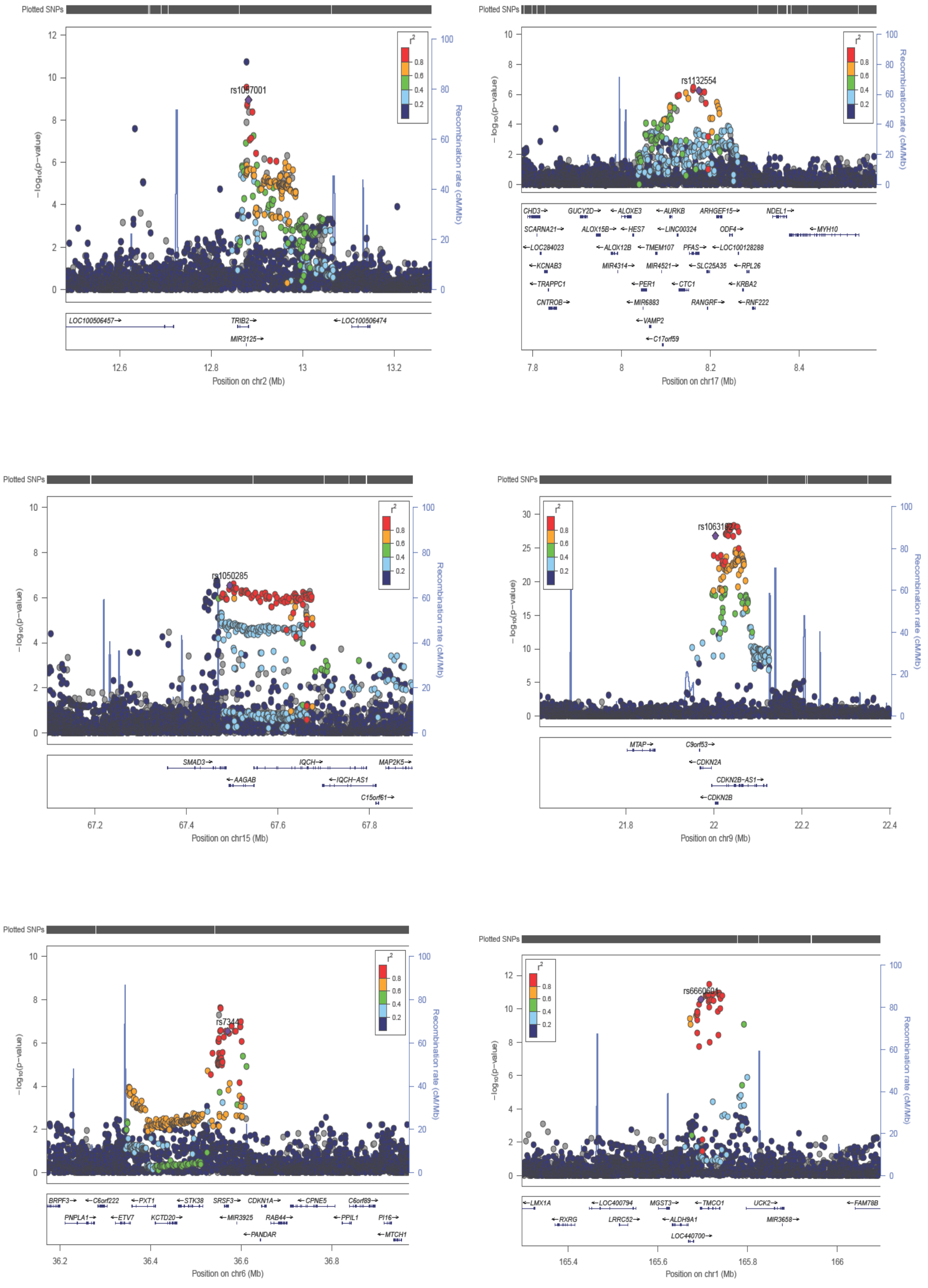

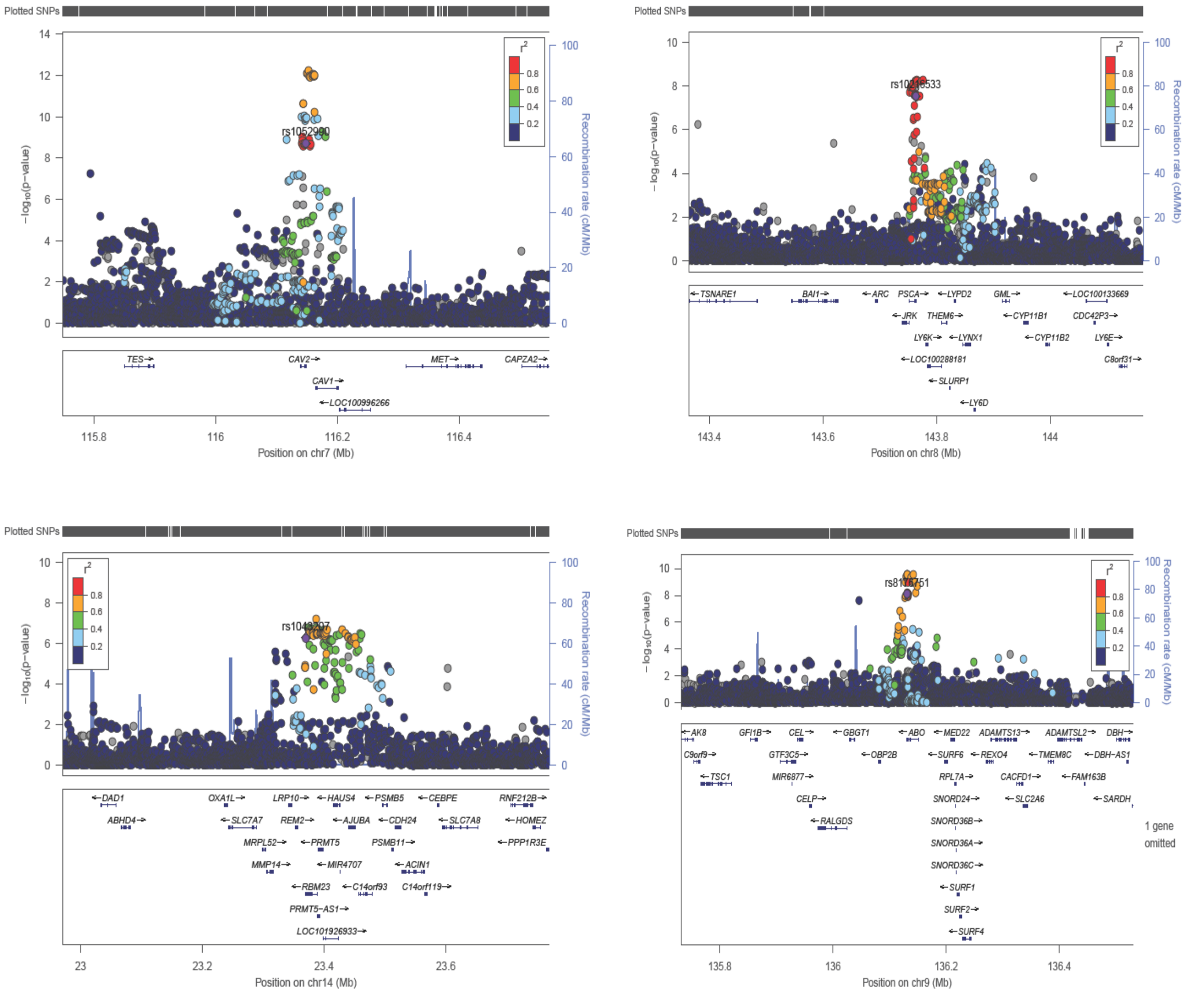

Plotted SNPs
−log10(p−value)
Recombination rate (cM/Mb)
r²
Position on chr7 (Mb)
TES
CAV2
CAV1
LOC100996266
MET
CAPZA2
Position on chr8 (Mb)
TSNARE1
BAI1
ARC
PSCA
LYPD2
GML
LOC100133669
JRK
THEM6
CYP11B1
CDC42P3
LY6K
LYNX1
CYP11B2
LY6E
LOC100288181
C8orf31
SLURP1
LY6D
Position on chr14 (Mb)
DAD1
OXA1L
LRP10
HAUS4
PSMB5
CEBPE
RNF212B
ABHD4
SLC7A7
REM2
AJUBA
CDH24
SLC7A8
HOMEZ
MRPL52
PRMT5
PSMB11
PPP1R3E
MMP14
MIR4707
ACIN1
RBM23
C14orf93
C14orf119
PRMT5−AS1
LOC101926933
Position on chr9 (Mb)
AK8
GFI1B
CEL
GBGT1
ABO
MED22
ADAMTS13
ADAMTSL2
DBH
C9orf9
GTF3C5
OBP2B
SURF6
REXO4
TMEM8C
DBH−AS1
TSC1
MIR6877
RPL7A
CACFD1
FAM163B
CELP
SNORD24
SLC2A6
SARDH
RALGDS
SNORD36B
SNORD36A
SNORD36C
SURF1
SURF2
SURF4
1 gene omitted
